# Talin and vinculin combine their activities to trigger actin assembly

**DOI:** 10.1038/s41467-024-53859-1

**Published:** 2024-11-03

**Authors:** Hong Wang, Rayan Said, Clémence Nguyen-Vigouroux, Véronique Henriot, Peter Gebhardt, Julien Pernier, Robert Grosse, Christophe Le Clainche

**Affiliations:** 1 Université Paris-Saclay, CEA, CNRS, Institute for Integrative Biology of the Cell (I2BC), Gif-sur-Yvette, France; 2 Institute of Experimental and Clinical Pharmacology and Toxicology, Medical Faculty, University of Freiburg, Freiburg, Germany; 3 Centre for Integrative Biological Signalling Studies-CIBSS, University of Freiburg, Freiburg, Germany

**Keywords:** Cytoskeletal proteins, Kinetics, Biochemical assays

## Abstract

Focal adhesions (FAs) strengthen their link with the actin cytoskeleton to resist force. Talin-vinculin association could reinforce actin anchoring to FAs by controlling actin polymerization. However, the actin polymerization activity of the talin-vinculin complex is not known because it requires the reconstitution of the mechanical and biochemical activation steps that control the association of talin and vinculin. By combining kinetic and binding assays with single actin filament observations in TIRF microscopy, we show that the association of talin and vinculin mutants, mimicking mechanically stretched talin and activated vinculin, triggers a sequential mechanism in which filaments are nucleated, capped and released to elongate. In agreement with these observations, FRAP experiments in cells co-expressing the same constitutive mutants of talin and vinculin revealed accelerated growth of stress fibers. Our findings suggest a versatile mechanism for the regulation of actin assembly in FAs subjected to various combinations of biochemical and mechanical cues.

## Abstract (web summary)

In cells, focal adhesions (FAs) strengthen their connection with the actin cytoskeleton to resist force. By combining biochemistry and cell biology, the authors show that the proteins talin and vinculin control actin assembly, thus reinforcing the anchoring of actin to FAs.

## Introduction

To migrate efficiently in different tissues, cells must sense and adapt to variations in the mechanical properties of their environment. In this adaptive process, focal adhesions (FAs) can strengthen their link with the extracellular matrix (ECM) and the actomyosin stress fibers^1–3^. FAs are composed of transmembrane integrins that mechanically couple the ECM to the actomyosin cytoskeleton, via a variety of actin binding proteins (ABPs)^2,4–6^.

The mechanical coupling of stress fibers to FAs is highly regulated. The anchoring of actin filaments to FAs can be modulated by the degree of engagement of a molecular clutch composed of sliding layers of interacting proteins, including ABPs^1,7^. The regulation of the polymerization of the actin filaments that compose the stress fibers may also determine the level of their mechanical coupling to FAs^2^. Interestingly, FAs associated with elongating dorsal stress fibers are associated with low traction forces, whereas FAs associated with slowly elongating ventral stress fibers are associated with high traction forces^8^. This inverse correlation between the elongation of the actin filaments and the transmission of force to the ECM sheds light on the importance of force-dependent ABPs which control actin assembly in FAs.

Biochemical and cellular studies have described a variety of ABPs associated with FAs that regulate the elongation of actin filament barbed ends. Early in vitro studies showed that the vasodilator-stimulated phosphoprotein (VASP) nucleates actin filaments and assembles them into bundles^9^. More recent studies demonstrate that VASP can also elongate actin filament barbed ends in a processive manner^10–12^. Similarly, a series of studies suggested that formins are involved in the processive elongation of stress fibers in cells^13,14^. Talin and vinculin, which associate in response to the actomyosin force, could link force sensing to the control of actin polymerization in FAs.

Vinculin is an autoinhibited ABP in which the single actin-binding domain (ABD), called vinculin tail (V_t_), is masked by an intramolecular interaction with vinculin head (V_h_)^15^. Biochemical studies showed that isolated V_t_ binds actin filaments and assembles them into bundles^16,17^. We showed that V_t_ also caps actin filament barbed ends and nucleates actin filaments^18^. All these activities of V_t_ are masked by V_h_ in the autoinhibited conformation of vinculin.

Talin is a large ABP composed of a FERM domain, subdivided into F0, F1, F2, F3, and a rod domain made of 13 helical bundles (R1 to R13)^2^. The two major intramolecular interactions F3-R9 and F2-R12 keep talin in an autoinhibited form^19,20^. Talin contains three ABDs. ABD1, ABD2 and ABD3 correspond to the head (F2F3), the central region (R4-R8) and the last C-terminal bundle (R13) respectively^21^. We previously demonstrated that the N-terminal ABD1 of talin blocks the elongation of actin filament barbed ends in low ionic strength conditions, whereas ABD2 and ABD3 do not affect actin dynamics^22^. Full-length talin is inactive because ABD1 is inhibited by the F3-R9 intramolecular interaction.

Biochemical and structural studies revealed the presence of 11 vinculin binding sites (VBSs) buried in some of the 13 helical bundles of talin^23^. The stretching of single molecules of talin revealed that force exposes several of these 11 cryptic VBSs along talin rod domain^24–26^. Using an in vitro reconstitution approach, we demonstrated that the actomyosin force is sufficient to stretch talin, allowing its binding to vinculin^27^. The release of talin autoinhibition also favors the low-affinity constitutive binding of vinculin^28^.

In order for the V_h_ domain of vinculin to interact with talin VBSs, it is necessary to release it from its autoinhibitory interaction with V_t_^29^. Although the mechanism of activation of vinculin is not fully understood, several studies show that once vinculin is bound to talin, the interaction of V_t_ with actomyosin keeps vinculin under tension in its open conformation^30–34^. In this mechanism, the actomyosin-dependent talin-vinculin complex acts as a catch-bond that releases V_t_ to strengthen the anchoring to actomyosin whose contraction initially induced its formation^27,34,35^.

Because force is required to trigger vinculin association to talin, and because the two proteins are autoinhibited, it has so far been impossible to determine the ability of the talin-vinculin complex to regulate actin polymerization.

Here, we use a series of talin and vinculin mutants that associate constitutively into a stable complex. By combining kinetic studies in fluorescence spectroscopy, actin binding assays, single actin filament observations in TIRF microscopy and FRAP experiments in cells, we show how these mutants and their complexes interact with actin filaments and regulate their polymerization in vitro, and control the elongation of stress fibers in cells. Altogether our data reveal a force-dependent mechanism of actin assembly in FAs.

## Results

### The release of the two autoinhibitory contacts of vinculin allows F-actin binding and barbed-end capping but not nucleation

**Fig. 1:**
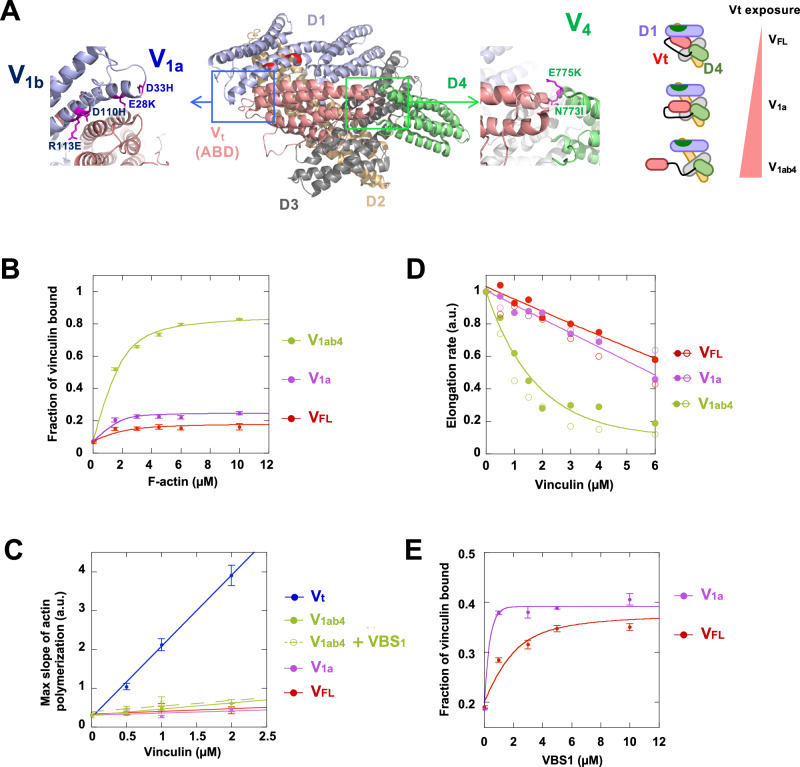
The release of the autoinhibitory contacts of vinculin allows F-actin binding and barbed-end capping but not nucleation. **A** Structure of vinculin featuring the subdomains and the auto-inhibitory contacts D1-V_t_ (blue box) and D4-V_t_ (green box). In D1, the double mutations E28K/D33H and D110H/R113E are referred to as V_1a_ and V_1b_ respectively. The double mutation N773I/E775K in D4 is referred to as V_4_. The mutated amino acids are in magenta. A schematic representation of the vinculin mutants used in this study is shown on the right. We used the existing structure PDB:1TR2 (https://www.rcsb.org/structure/1tr2). **B** Quantification of the co-sedimentation of the indicated vinculin mutants (2 µM) in the presence of increasing concentration of F-actin. The fraction of vinculin bound in the pellet is plotted against F-actin concentration (Supplementary Fig. 1). **C** The maximal rate of spontaneous actin polymerization (1.5 µM, 10% pyrenyl-labeled) is plotted against the concentration of the indicated vinculin mutants (Supplementary Fig. 2). V_t_ is used as a positive control for nucleation. **D** Actin filament barbed end elongation is measured in the presence of spectrin-actin seeds (100 pM), 2 µM actin (10% pyrenyl-labeled), and increasing concentrations of the indicated vinculin mutants (Supplementary Fig. 3). The fraction of barbed end elongation is the ratio between the elongation rates in the presence and absence of vinculin. Open and closed circles indicate two independent experiments in the same conditions. **C**, **D** Actin polymerization and elongation rates are expressed in arbitrary units (a.u.). **E** The binding of the indicated vinculin constructs (2 µM) to F-actin (10 µM) is measured in a co-sedimentation assay in the presence of increasing concentrations of talin VBS_1_ (Supplementary Fig. 5). The fraction of vinculin bound to F-actin is plotted against the concentration of VBS_1_. In (**B**–**E**), data are mean ± SE, three independent experiments. The curves are not fits of the experimental points by a model, but are drawn manually to reflect the trend of the data, in order to make the data easier to read. Source data are provided as a Source Data file.

Before starting the study of the talin-vinculin complex, we deciphered the complex mechanism that links the autoinhibition of vinculin and its activities. Indeed, biochemical and structural studies have revealed that the auto-inhibition of vinculin is controlled by two interfaces between V_t_ and the D1 and D4 subdomains of the head^18,36,37^ (Fig. 1A).

In order to disrupt specifically both autoinhibitory interfaces, we designed a series of point mutations of amino acids in D1 and D4 that interact with V_t_. Because the contact surface between V_t_ and D1 is significantly larger than the D4-V_t_ one, we mutated two separate groups of charged amino acids in the D1-V_t_ interface: E28K, D33H referred to as V_1a_ and D110H, R113E referred to as V_1b_ (Fig. 1A). Their combination is referred to as V_1ab_. In D4, we designed a double mutation N773I, E775K called V_4_ (Fig. 1A). In most of our comparative studies, we used full-length vinculin (V_FL_), the isolated ABD V_t_ and the V_1a_ and V_1ab4_ mutants.

We first characterized these vinculin mutants by measuring their binding to actin filaments (F-actin) using a cosedimentation assay. As previously reported, the autoinhibited V_FL_ has low affinity for F-actin^18^ (Fig. 1B, Supplementary Fig. 1A). Among the mutants, V_1ab4_ displays a high affinity for F-actin (Fig. 1B and Supplementary Fig. 1B), while V_1a_ displays low to intermediate binding level (Fig. 1B and Supplementary Fig. 1C). Altogether, these data demonstrate that the combined release of D4 and D1 efficiently exposes V_t_ (Supplementary Table 1).

We then tested the effect of these vinculin mutants on actin nucleation by measuring the acceleration of the kinetics of pyrenyl-labeled actin assembly in low ionic strength condition, which corresponds to 25 mM KCl, while the salt concentration normally used by the vast majority of studies is 50 mM KCl and sometimes 100 mM KCl. Several studies have revealed regulatory mechanisms and activities of FA proteins, such as vinculin, talin and VASP, by varying the ionic strength of in vitro assays^9,18,20^. We first confirmed that V_t_ nucleates actin filament in a dose-dependent manner^18^ (Fig. 1C and Supplementary Fig. 2A). In contrast, V_FL_, V_1a_, and V_1ab4_ do not show significant actin nucleation activities (Fig. 1C and Supplementary Fig. 2B-D, Supplementary Table 1). Although the activity is very weak, the kinetics showed that V_1ab4_ displays a measurable nucleation activity (Supplementary Fig. 2D), suggesting that the release of V_t_ from both D1 and D4 is necessary to nucleate actin, but not sufficient to fully expose this activity of V_t_.

These two series of experiments lead to an apparent contradiction, since the release of the 2 contacts D1-V_t_ and D4-V_t_ is sufficient to expose V_t_ and allow it to bind to F-actin (Fig. 1B), but not to nucleate actin filaments (Fig. 1C). We hypothesized that, in addition to releasing the D1-V_t_ and D4-V_t_ contacts, F-actin, which is present in cosedimentation assays, but absent at the beginning of the nucleation assays, contributes to the opening of vinculin by binding to V_t_. To test this hypothesis, we compared the ability of vinculin mutants to cap preexisting actin filament barbed ends. The effect of vinculin mutants on barbed end capping was first assessed by measuring the inhibition of pyrenyl-labeled actin polymerization in the presence of spectrin-actin seeds^18^. Although V_FL_ and V_1a_ display only weak activity (Fig. 1D, Supplementary Fig. 3A, B), V_1ab4_ strongly inhibits the elongation of preexisting actin filament barbed ends, in agreement with our hypothesis (Fig. 1D, Supplementary Fig. 3C, and Supplementary Table 1).

### An isolated vinculin-binding domain of talin promotes vinculin binding to F-actin but has no effect on nucleation

Before determining the activity of the talin-vinculin complex, it was necessary to determine whether and how the simple binding of talin to vinculin, independently of the activities of talin ABDs, influences the activities of vinculin. We therefore used a short domain of talin, called VBS_1_ (talin 482-636) corresponding to R1 deleted from its last helix, which exposes one VBS^18^ (Supplementary Fig. 4).

First, VBS_1_ does not increase the very weak nucleation activity of V_1ab4_, which remains negligible compared to that of V_t_ (Fig. 1C and Supplementary Fig. 2A, E). This assay, in which F-actin is initially absent, further supports the importance of F-actin as a co-activator of vinculin.

We then tested the effect of VBS_1_ (0–10 µM) on the ability of vinculin to bind to F-actin, using a cosedimentation assay (Fig. 1E and Supplementary Fig. 5). VBS_1_ induces an increase in V_FL_ binding to F-actin (Fig. 1E and Supplementary Fig. 5A). The weakening of D1-V_t_ increases the effect of VBS_1_, which results in a higher apparent affinity of V_1a_ for F-actin (Fig. 1E and Supplementary Fig. 5B).

### Talin with exposed VBSs promotes vinculin-dependent barbed-end capping but has no effect on nucleation

After determining the contribution of an isolated talin VBS to the activity of vinculin, we designed a series of full-length talin constructs (TΔ1, TΔ2, TΔ3) in which VBSs are exposed (Supplementary Figs. 6A and 4), in order to study the activity of a constitutive talin-vinculin complex harboring the ABDs of both proteins. We first designed a construct, referred to as TΔ1, in which the helix 5 of the helical bundle R1 is deleted, which exposes the same VBS than that of the construct called VBS_1_ used in Fig. 1. Talin TΔ2 has been designed to expose two consecutive VBSs in R3, thanks to the deletion of the 2 helices 10 and 13 located on both sides of the two VBSs. Talin TΔ3 combines the mutations of TΔ1 and TΔ2. We first verified the efficiency of deletions performed in TΔ1, TΔ2, and TΔ3 using a micropatterning-based binding assay adapted from our previous work^27,38,39^. Hence, TΔ1, TΔ2, TΔ3 constructs immobilized on micropatterned surfaces recruit the head of vinculin fused to EGFP (V_h_-EGFP) more efficiently than the wild-type T_FL_ (Supplementary Fig. 6B, C). Because it is impossible to distinguish the relative contribution of each protein in the talin-vinculin complex to F-actin binding, we did not perform cosedimentation assays for this part of the study.

We then determined the contribution of talin to the barbed-end capping activity of the talin-vinculin complex. To this aim, we combined the talin mutants with the V_1a_ mutant of vinculin which does not efficiently cap actin barbed ends alone (Fig. 1D). In the presence of a fixed concentration of V_1a_, addition of increasing concentrations of TΔ1, TΔ2 and TΔ3 induces a dose-dependent inhibition of barbed-end elongation in a spectrin-actin seed assay (Supplementary Fig. 6D, Supplementary Fig. 7B–D, and Supplementary Table 1). Wild-type full-length talin (T_FL_) also induces barbed-end inhibition in the presence of V_1a_, but with a lower efficiency than TΔ1, TΔ2 and TΔ3 (Supplementary Fig. 6D, Supplementary Fig. 7A, and Supplementary Table 1). This effect is not due to talin constructs alone and requires the presence of V_1a_ since T_FL_, TΔ1, TΔ2, and TΔ3 alone had no effect on barbed end elongation (Supplementary Figs. 6D and 8A–D). The fact that the fully autoinhibited V_FL_ does not combine with T_FL,_ TΔ1, TΔ2, and TΔ3 to inhibit barbed end elongation efficiently confirms that vinculin activation is required for this activity (Supplementary Fig. 9A–E).

We then tested the ability of the talin-vinculin complex to stimulate actin assembly by combining the talin mutants with the vinculin mutant V_1ab4_. The mutant V_1ab4_ appeared to be the ideal choice here because it is close to the fully open conformation (Fig. 1A). We hypothesized that the weak nucleation activity of V_1ab4_, (Fig. 1C and Supplementary Fig. 2D), could be increased by talin binding to vinculin head and the activity of talin ABDs. However, kinetic assays containing V_1ab4_ and T_FL,_ TΔ1, TΔ2, or TΔ3, do not reveal a significant nucleation activity compared to the activity of V_t_ alone, and it is not higher than that of V_1ab4_ alone (Supplementary Figs. 6E and 10A–D, and Supplementary Table 1).

### A talin-vinculin complex, in which all autoinhibitory contacts are released, nucleates actin filaments transiently capped at their barbed ends

**Fig. 2:**
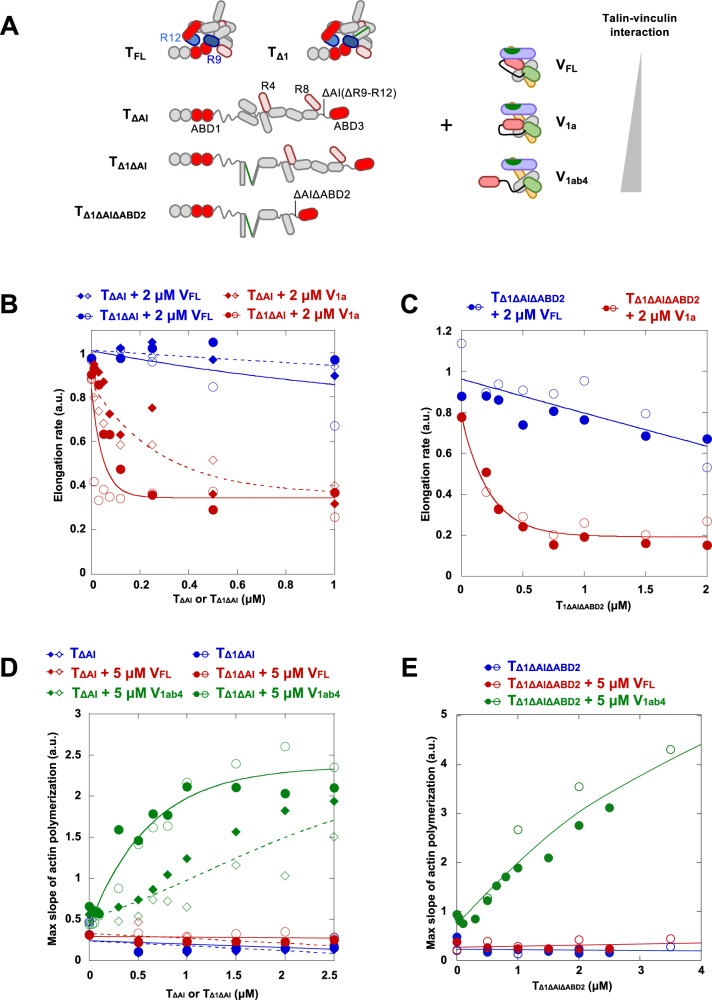
A complex of talin and vinculin, in which all autoinhibitory contacts are released and VBSs are exposed, nucleates and caps actin filaments. **(A)** Schematic representation of the talin and vinculin constructs used in (**B**–**E**). **B**, **C** Actin filament barbed end elongation, expressed in arbitrary units (a.u.), is measured in the presence of 100 pM spectrin-actin seeds, 1.5 µM actin (10% pyrenyl-labeled), 2 μM V_FL_ (blue) or 2 µM V_1a_ (red) and increasing concentrations of (**B**) T_ΔAI_ (dash line) or T_Δ1ΔAI_ (solid line), or (**C**) T_Δ1ΔAIΔABD2_. The fraction of barbed end elongation was then calculated as the ratio between the elongation rate in the presence of the indicated combinations of vinculin and talin and the elongation rate of actin alone. **D**, **E** The maximal rate of spontaneous actin polymerization (2.5 µM, 10% pyrenyl-labeled), expressed in arbitrary units (a.u.), is plotted as a function of increasing concentrations of (**D**) T_ΔAI_ or T_Δ1ΔAI_, (**E**) T_Δ1ΔAIΔABD2_ alone and in the presence of V_FL_ or V_1ab4_. In **B**–**E**, open and closed symbols indicate two independent experiments in the same conditions. The curves are not fits of the experimental points by a model, but are drawn manually to reflect the trend of the data, in order to make the data easier to read. Source data are provided as a Source Data file.

The results obtained with the previous talin mutants may only partially reflect the activity of the talin-vinculin complex because talin remains autoinhibited by the F3-R9 and F2-R12 contacts^19,20^. We previously demonstrated that the F3-R9 contact masks the capping activity of talin ABD1^22^. Therefore, we hypothesized that releasing these autoinhibitory contacts in talin may unravel additional activities of the talin-vinculin complex. To test this hypothesis, we designed three talin constructs, called TΔAI, TΔ1ΔAI and TΔ1ΔAIΔABD2 (Fig. 2A and Supplementary Fig. 4). TΔAI corresponds to T_FL_ deleted from the region encompassing the auto-inhibitory (AI) modules R9 and R12. TΔ1ΔAI combines the deletion of the AI region (ΔAI) and the deletion that exposes a VBS in R1 (Δ1). TΔ1ΔAIΔABD2 combines the ΔAI and Δ1 deletions with the deletion of the second actin binding domain (ΔABD2).

In a spectrin-actin seed assay, increasing concentrations of TΔAI and TΔ1ΔAI stimulate the barbed-end capping activity of V_1a_ but not that of V_FL_ (Fig. 2B, Supplementary Fig. 11A–E, and Supplementary Table 1). Altogether these experiments show that releasing the autoinhibitions of talin favors its association with vinculin to form a complex that caps actin filament barbed ends, provided that vinculin autoinhibition is also weakened. The fact that TΔ1ΔAI is much more effective than TΔAI for this activity further indicates that the release of the autoinhibition of talin adds to the VBS exposure in R1 to promote vinculin binding. The strong barbed-end capping activity of the complex made of TΔ1ΔAIΔABD2 and V_1a_, but not V_FL_, also indicates that talin ABD2 is not required for this activity (Fig. 2C, Supplementary Fig. 12A–C, and Supplementary Table 1). The number and position of exposed VBSs influence the barbed end capping activity of the complex since TΔ2ΔAI, in which two VBSs are exposed in R3, and TΔ3ΔAI, in which three VBSs are exposed in R1 and R3, combine efficiently with V_FL_ to cap actin filaments (Supplementary Fig. 13A–E and Supplementary Table 1), in contrast with TΔ1ΔAI, in which only one VBS is exposed in R1 (Fig. 2B).

To confirm that this inhibition of elongation is indeed due to an interaction with the barded end, we compared the ability of the TΔ1ΔAI-V_1a_ complex to inhibit the elongation of spectrin-actin seeds and the elongation mediated by the FH1-FH2 fragment of the formin mDia1, which is known to elongate filaments while residing processively at their growing barded end, thus protecting the filaments from barbed-end capping. Our quantifications show that actin filaments polymerizing in the presence of mDia1 are less effectively inhibited by the TΔ1ΔAI-V_1a_ complex than filaments with free barbed ends, confirming the binding of the complex to the barbed end (Supplementary Fig. 14A–C).

We then tested the effect of complexes composed of TΔAI, TΔ1ΔAI, and TΔ1ΔAIΔABD2 and vinculin on actin nucleation. Increasing concentrations of TΔAI and TΔ1ΔAI stimulate actin assembly in the presence of V_1ab4_ but not in the presence of V_FL_ (Fig. 2D, Supplementary Fig. 15A–D, and Supplementary Table 1). As for the capping activity (Fig. 2B), TΔ1ΔAI is much more effective than TΔAI for the nucleation activity, indicating that, in addition to the release of talin autoinhibition, VBS exposure is required for talin and vinculin to form a nucleation complex (Fig. 2D and Supplementary Fig. 15C, D). The number and position of exposed VBSs influence this activity of the complex since a complex made of V_1ab4_ and TΔ3ΔAI, in which three VBSs are exposed in R1 and R3, is slightly more active than complexes made of V_1ab4_ and TΔ1ΔAI or TΔ2ΔAI, in which one or two VBSs are exposed respectively. This effect is visible at high concentration of TΔ3ΔAI (Supplementary Fig. 16A–F and Supplementary Table 1).

This nucleation activity is strong at 25 mM KCl and remains significant at 50 mM KCl but disappears at 75 mM KCl, indicating the electrostatic nature of interactions between talin, vinculin and actin (Supplementary Fig. 17A, B). The conditions we used to measure the stimulation of actin assembly by talin and vinculin are close to those generally used to study actin polymerization activities in vitro with purified proteins. However, these conditions (20 °C, pH = 7.8, 25 mM KCl) are not physiological. We therefore performed kinetic assays under physico-chemical conditions as close as possible to those found in mammalian cells, i.e. 37 °C, pH = 7, 100 mM KCl. Under these conditions, spontaneous actin polymerization is faster and the stimulation of actin polymerization by the combined action of TΔ1ΔAI or TΔ3ΔAI and V_1ab4_ is strong (Supplementary Fig. 18A, B). Importantly, in cells, polymerizable actin is complexed with profilin, which prevents spontaneous nucleation of actin filaments and restricts the formation of new filaments at specific sites, generally associated with a membrane-bound structure stimulated by chemical or mechanical signals. We therefore tested the effect of profilin on nucleation activity. Although profilin does not completely abolish this activity, the very strong reduction observed suggests that an additional mechanism is involved in the dissociation of profilin (Supplementary Fig. 18B).

The fact that combinations of activated vinculin V_1ab4_ with auto-inhibited talin (TΔ1, TΔ2, and TΔ3) do not stimulate actin assembly indicates that autoinhibited ABDs in talin play a critical role in this mechanism. To determine the relative importance of the ABDs of talin and vinculin for the activity of the complex, we tested various constructs of the two proteins deleted for ABDs and we added competitors of ABDs to polymerization reactions. First, we showed that talin ABD2 is not necessary since TΔ1ΔAIΔABD2, lacking ABD2, efficiently combines with V_1ab4_ to stimulate actin assembly (Fig. 2E, Supplementary Fig. 19A–C, and Supplementary Table 1). We also compared the ability of talin constructs containing VBSs and either ABD1 or ABD3 to stimulate actin assembly in the presence of V_1ab4_. We found that ABD1 and ABD3 can combine with vinculin to stimulate actin polymerization, suggesting redundancy between ABD1 and ABD3 (Supplementary Fig. 20A). Interestingly, the addition of the isolated R9 domain of talin to a polymerization reaction containing TΔ1ΔAIΔABD2 and V_1ab4_ reduces the stimulation of actin assembly (Supplementary Fig. 20B). This result confirms that ABD1, which is masked by R9 in inactive talin^22^, is involved in this activity. Finally, combinations of various activated talin constructs with vinculin head (V_h_), which lacks its ABD V_t_, do not block the elongation of actin filament barbed ends or stimulate actin assembly, indicating a critical role of V_t_ in all activities of the talin-vinculin complex (Supplementary Fig. 21A, B). Altogether our observations indicate that ABD1 and ABD3 domains of talin, as well as the V_t_ domain of vinculin, are involved in the activities of the complex.

To confirm the mechanism by which the talin-vinculin complex regulates actin assembly, we observed in TIRF microscopy the actin filaments produced by the complex made of V_1ab4_ and TΔ1ΔAI or TΔ1ΔAIΔABD2 described above.

**Fig. 3:**
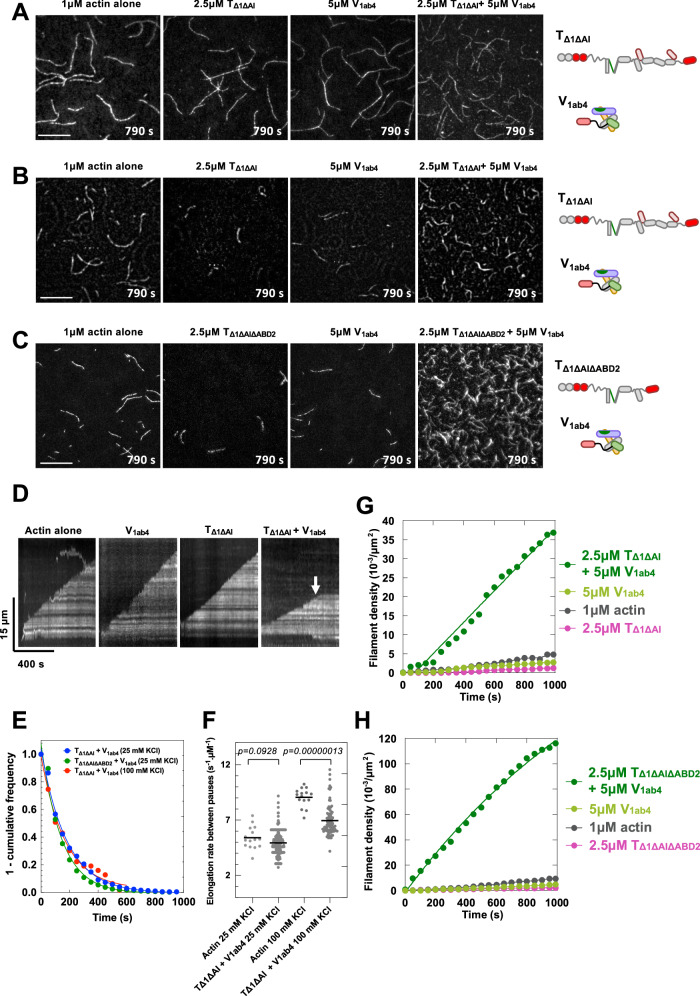
Direct observation of the nucleation and capping of single actin filaments by the talin-vinculin complex. **A**–**C** Single actin filaments observed in TIRF microscopy in the presence of 1 µM actin (5% Alexa488-labeled) alone and supplemented with 2.5 µM T_Δ1ΔAI_ alone, 5 µM V_1ab4_ alone and 2.5 µM T_Δ1ΔAI_ with 5 µM V_1ab4_ at 100 mM KCl (**A**) or 25 mM KCl (**B**), and in the presence of 1 µM actin (5% Alexa488-labeled) alone and supplemented with 2.5 µM T_Δ1ΔAIΔABD2_ alone, 5 µM V_1ab4_ alone and 2.5 µM T_Δ1ΔAIΔABD2_ with 5 µM V_1ab4_ at 25 mM KCl (**C**). Scale bar = 15 μm. See Supplementary Movie 1 (**A**), Supplementary Movie 2 (**B**), and Supplementary Movie 3 (**C**). Experiments in (**A**–**C**) were repeated twice independently with the same results. **D** Kymographs of single actin filaments in the presence of the indicated proteins from the experiment in (**A**). The arrow indicates a barbed end capping event. **E** Exponential fit of 1-cumulative frequency of barbed end pause times measured in the presence of 1 µM actin, 2.5 μM T_Δ1ΔAI_ with 5 μM V_1ab4_ (red line at 100 mM KCl, *t*_1/2_ = 115.8 ± 22 s, *n* = 349, blue line at 25 mM KCl, *t*_1/2_ = 124.2 ± 10.8 s, *n* = 1023) and 2.5 μM T_Δ1ΔAIΔABD2_ with 5 μM V_1ab4_ (green line, *t*_1/2_ = 99.6 ± 13.5 s, *n* = 1619) from experiments in (**A**–**C**). **F** Quantification of the elongation rates of single filaments between pauses in the experiments shown in (**A**), *n* = 15 for actin alone, *n* = 263 for T_Δ1ΔAI_ + V_1ab4_ and (**B**), *n* = 15 for actin alone, *n* = 91 for T_Δ1ΔAI_ + V_1ab4_. Data were analyzed using a two-sided unpaired t-test. **G**, **H** Quantification of filament density as a function of time from the experiments in (**B**) and (**C**). **A** is not quantified as nucleation is weak at 100 mM KCl. Source data are provided as a Source Data file.

At high salt concentration (100 mM KCl), which restricts the activity of the talin-vinculin complex to the capping of the barbed ends only, the filaments show pauses interrupting the elongation of their barbed end (Fig. 3A, D and Supplementary Movie 1). Quantifying the distribution of these pause times enabled us to estimate a dissociation rate constant of the complex from the barbed end of 0.006 s^−1^ (*t*_1/2_ = 115.8 s) (Fig. 3E). Quantification of the elongation rate between pauses revealed a slight reduction in the presence of V_1ab4_ and TΔ1ΔAI reflecting the presence of remaining short capping events that escaped our analysis or an effect on the elongation other than barbed end capping (Fig. 3F). In low salt concentration, favoring actin nucleation by the talin-vinculin complex (Fig. 2D, E), representative images and quantifications showed that the TΔ1ΔAI-V_1ab4_ complex induces the formation of a higher number of actin filaments than TΔ1ΔAI alone or V_1ab4_ alone (Fig. 3B, G and Supplementary Movie 2). We found the same result for TΔ1ΔAIΔABD2-V_1ab4_ complex (Fig. 3C, H and Supplementary Movie 3). The analysis of actin filament elongation showed that they undergo frequent pauses reflecting barbed end capping events. The exponential fit of the distribution of the pause duration gave an estimated dissociation rate constant of 0.0056 s^−1^ (*t*_1/2_ = 124.2 s) for TΔ1ΔAI-V_1ab4_ and 0.007 s^−1^ (*t*_1/2_ = 99.6 s) for TΔ1ΔAIΔABD2-V_1ab4_ (Fig. 3E). Quantification of the elongation rate between pauses did not reveal any effect of V_1ab4_ and TΔ1ΔAI (Fig. 3F).

**Fig. 4:**
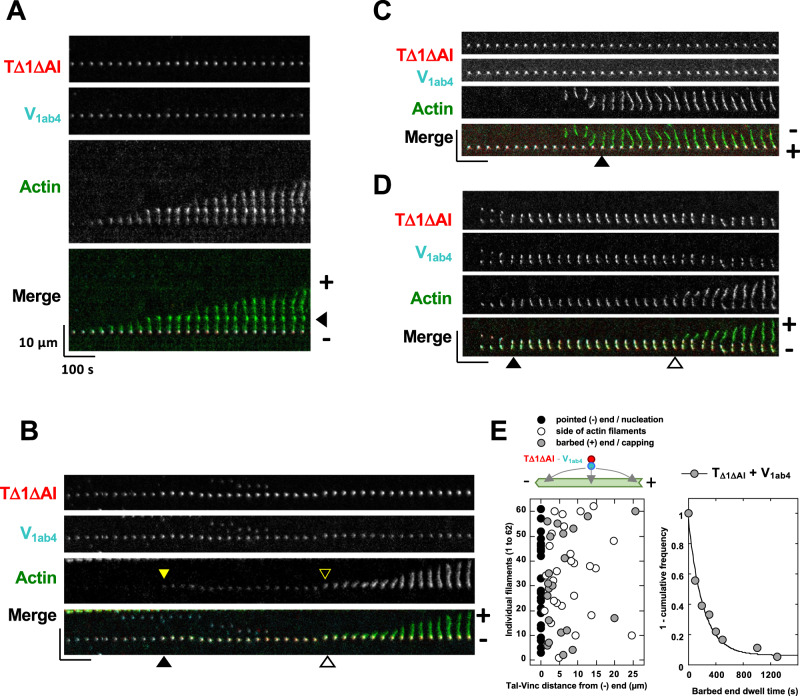
Direct observation of talin, vinculin and actin during the nucleation and barbed-end capping of single actin filaments. **A**–**E** 10 nM TΔ1ΔAI (78% Alexa594-labeled) and 50 nM V_1ab4_ (18% Alexa647-labeled) are immobilized on a glass surface passivated with PLL-PEG, followed by the addition of 0.8 µM actin (5% Alexa488-labeled). **A** Kymographs showing the nucleation of an actin filament by a talin-vinculin complex. The stationary fiduciary mark indicated by the arrowhead on the right side indicate that the talin-vinculin complex remains associated near the pointed end (−) while the barbed end (+) grows on the other side (Supplementary Movie 4). **B** Recruitment of actin by a talin-vinculin complex followed by the elongation of a filament after a delay indicated by the arrowheads (Supplementary Movie 5). The delays are quantified in (**E**). **C** Kymographs showing the capture and barbed-end capping of an existing filament by a talin-vinculin complex (Supplementary Movie 6). **D** Kymographs showing the transient barbed-end capping of a filament by a talin-vinculin complex followed by its release (Supplementary Movie 7). Experiments in (**A**–**D**) were repeated twice independently with the same results. **E** (Left) Position of talin-vinculin complexes measured along 62 single filaments aligned on their pointed end (−). This quantification reveals that talin-vinculin complexes are found near the pointed end of newly nucleated filaments (black dots, *n* = 22), along the side of actin filaments (white dots, *n* = 26) or near the barbed end of capped filaments (gray dots, *n* = 21). Some of these events are on the same filaments. (Right) Exponential fit of 1-cumulative frequency of the time between actin recruitment by talin-vinculin complexes and barbed end elongation measured in the conditions described above and illustrated in (**B**), *t*_1/2_ = 146.69 ± 18.95 s, *n* = 18. Source data are provided as a Source Data file.

To explore the mechanisms underlying the above microscopy observations, we followed fluorescent molecules of vinculin, talin and actin in real time during the nucleation and capping of individual actin filaments. We first immobilized pre-formed complexes of TΔ1ΔAI-Alexa594 and V_1ab4_-Alexa647 at low concentration on a glass surface passivated with PLL-PEG and added actin-Alexa488 under conditions that allowed nucleation and capping. This experimental setup allowed us to capture sequences of events in which spots, containing both talin and vinculin, associate with actin and release a growing actin filament (Fig. 4A, Supplementary Movie 4). In this experiment, as in FAs that form in cells, talin, and vinculin are dimers that assemble with high stoichiometry. The observed spots therefore contain several molecules of TΔ1ΔAI and V_1ab4_ that assemble via the exposed VBS of TΔ1ΔAI but also via other VBSs with lower affinity as indicated by the activity of the TΔAI-V_1ab4_ experiments (Fig. 2D). The stationary fiduciary marks along actin filaments indicate that the talin-vinculin complex remains associated to the filament near the pointed end while the barbed end is free to grow on the other side (black arrow head, Fig. 4A). Interestingly, nucleation events are often characterized by the existence of a delay between actin recruitment by a talin-vinculin complex and the elongation of a filament (Fig. 4B and Supplementary Movie 5). In this series of experiments, pre-existing filaments are also captured and capped at their barbed end (Fig. 4C and Supplementary Movie 6). Barbed-end capping is transient, as many of the capped filaments are eventually left free to elongate after a delay (Fig. 4D and Supplementary Movie 7). These activities lead to a distribution of talin-vinculin complexes near the pointed end of the filaments, following their nucleation, at the barbed end of the filaments, following their capping, or on the side of the filaments (Fig. 4E). Quantification of the delay between actin recruitment and elongation allows to determine a rate (0.0047 s^−1^, *t*_1/2_ = 147 s, Fig. 4E), that is close to the dissociation rate of the barbed ends from the talin-vinculin complex observed previously (Fig. 3E), suggesting that the talin-vinculin complex nucleates filaments initially capped at their barbed end.

### The association of talin and vinculin contributes to the assembly of stress fibers in cells

To determine the effect of talin-vinculin complex formation on actin assembly in cells, we compared Hela cells co-expressing fluorescent wild-type talin and vinculin (BFP-Talin _FL_ and mCherry-Vinculin _FL_) with cells co-expressing BFP-TΔ1ΔAI and mCherry-V_1ab4_ mutants. We first quantified FA area labeled with mCherry-vinculin and the width of stress fibers labeled with Alexa488-phalloidin. Cells expressing TΔ1ΔAI and V_1ab4_ have larger FAs associated with wider stress fibers (Supplementary Fig. 22B, C, D), compared to cells expressing wild-type talin and vinculin (Supplementary Fig. 22A, C, D).

**Fig. 5:**
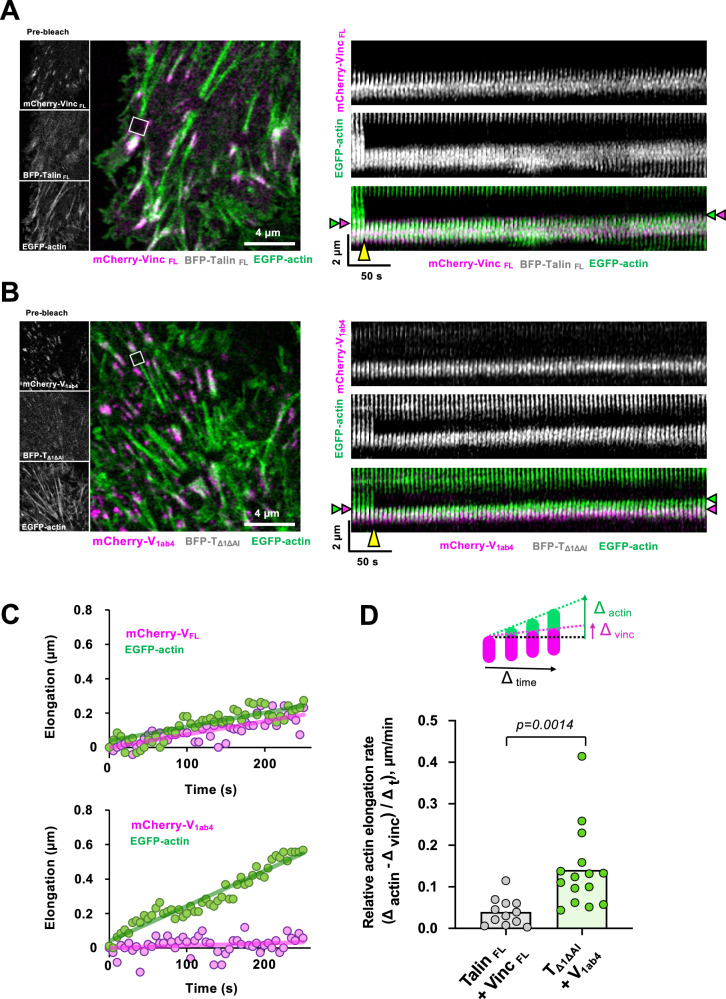
Constitutively active talin-vinculin complex accelerates stress fiber growth in Hela cells. **A**, **B** Representative images of Hela cells expressing EGFP-actin together with mCherry-Vinculin_FL_/BFP-Talin_FL_ (**A**) or mCherry-V_1ab4_/BFP-T_Δ1ΔAI_ (**B**). Expression of mCherrry-vinculin, BFP-talin and EGFP-actin before bleaching is shown on the smaller left panels. Expression of mCherry-vinculin and EGFP-actin together with the bleached area (white square) is shown in the larger left panels. The corresponding kymographs of bleached stress fibers are shown on the right. Note that BFP-talin, mCherry-vinculin and EGFP-actin have been imaged before bleaching and only mCherry-vinculin and EGFP-actin have been imaged after bleaching to reduce time intervals. **C** The apparent elongation of EGFP-actin and retrograde movement of m-Cherry-vinculin are plotted as a function of time in the conditions mentioned in (**A**) and (**B**). **D** Scatter plot showing the quantification of the elongation rate of actin from the edge of vinculin-labeled FA in the conditions mentioned in (**A**) and (**B**). FRAP experiments were performed on 6 cells expressing mCherry-vinculin_FL_ + BFP-talin_FL_ + EGFP-actin, *n* = 12 measurements (2, 2, 2, 2, 2, 2) from 5 independent experiments performed in 5 different days, and 5 cells expressing mCherry-V_1ab4_ + BFP-T_Δ1ΔAI_ + EGFP-actin, *n* = 15 measurements (5, 1, 1, 3, 5) from 4 independent experiments performed in 4 different days. Each measurement represents the elongation rate of a stress fiber subtracted from the rate of retrograde sliding of the associated FA. Data were analyzed using a two-sided unpaired t-test. Source data are provided as a Source Data file.

To determine whether the constitutive formation of the talin-vinculin complex enhances stress fiber assembly dynamics, we compared the rate at which EGFP-actin-containing stress fibers elongate by FRAP in Hela cells co-expressing wild-type BFP-talin and mCherry-vinculin, and in Hela cells co-expressing BFP-TΔ1ΔAI and mCherry-V_1ab4_ mutants (Fig. 5A, B and Supplementary Movie 8 and 9). As FAs slide retrogradely, we took this parameter into account and measured the rate of elongation of the stress fibers using the trailing edge of the FAs as a reference, revealed here by the fluorescence of mCherry-vinculin (Fig. 5C). Quantification of the relative actin-EGFP fluorescence recovery rate from the FAs indicates that stress fibers elongate faster in cells expressing TΔ1ΔAI and V_1ab4_ compared with cells expressing wild-type talin and vinculin (Fig. 5D). Taken together, our data indicate that binding of vinculin to talin in FAs leads to the stimulation of actin assembly.

## Discussion

The assembly of the talin-vinculin complex is a key step in the reinforcement of cell-matrix adhesions subjected to a mechanical stimulus such as the force generated by the actomyosin stress fibers. This complex strengthens the link with the tensile actomyosin cytoskeleton to resist the force it applies. However, the mechanism by which the force-dependent complex acts on actin filaments was poorly understood.

**Fig. 6:**
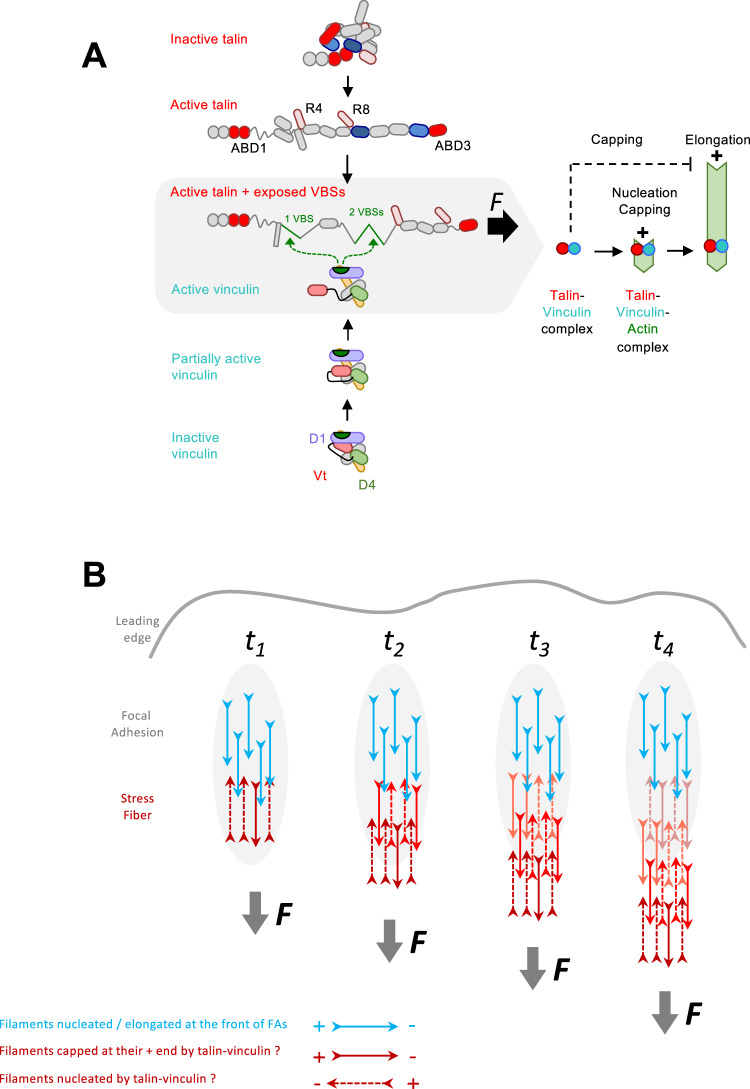
Working model for the activities and regulation of talin, vinculin and the talin-vinculin complex. **A** On the left side, talin (top) and vinculin (bottom) are shown according to their increasing degree of activation towards the central gray box in which the fully activated talin and vinculin form a complex. On the right side, a simplified representation shows the activity of the talin-vinculin complex nucleating an actin filament that is transiently capped at its barbed end before being released to elongate. **B** Scheme showing the known polarity of the actin filaments emerging from FAs by different colors depending on the orientation of their barbed end. The activities of the talin-vinculin complex that could explain these different orientations are indicated with the same color code.

The mechanistic study of the talin-vinculin complex has long been limited by the fact that the assembly of the complex depends on the release of multiple autoinhibitory interactions, both in talin and in vinculin. Although it is established that force exposes cryptic vinculin binding sites in talin by stretching helical bundles, the mechanisms allowing the release of the additional autoinhibitory interactions between distant domains in talin are not fully understood. The structure of full-length talin in its autoinhibited conformation has been reported and revealed that, in addition to the known interaction between F3 and R9, the F2 domain interacts with the R12 domain^19,20^. These autoinhibitory contacts mask the binding interface of ABD1, corresponding to F2 and F3, with F-actin, while ABD2, composed of R4 and R8, is masked by an interaction with R3^20,22,28^ (Fig. 6). The mechanism by which these contacts are disrupted is unclear. The binding of RIAM and PIP2 to talin F3 is known to disrupt the F3-R9 autoinhibition^40^. Once these two interdomain interactions are disrupted, talin can be pulled by actomyosin force, which stretches helical bundles to expose cryptic VBSs^24,25^ (Fig. 6). Force application to the ABD3/R13 domain of talin could also contribute to disrupting its auto-inhibitions, but no experimental evidence of such a mechanism has been reported to date. This mechanism is possible because the ABD3 domain is exposed, at least partially, in the inactive talin, as suggested by the flexibility of the neighboring domains^20^. In this study, we show that combining various deletions, including the part of talin that contains the R9 and R12 autoinhibitory domains, with the deletion of individual helices, which exposes VBSs, mimics the open and mechanically stretched conformation of talin (Fig. 6). Our results show that the release of talin autoinhibitions is sufficient to allow the binding of vinculin and form a complex that caps and nucleates actin, which explains the observation made by others that a mutation in talin that disrupts the F3-R9 interaction is sufficient to recruit vinculin in cells^28^. Our data also support the findings that the association of talin and vinculin without tension is required for efficient nascent adhesion maturation^41^. This interaction likely corresponds to a low degree of saturation of the partially exposed VBSs in this open talin structure that has become very flexible. Indeed, exposure of a single VBS motif in R1 significantly increases vinculin binding as evidenced by the enhanced activity of the talin-vinculin complex.

Vinculin is autoinhibited by an intramolecular interaction between the head (V_h_) and the tail (V_t_) domains. Talin binding to the D1 subdomain of V_h_ is controlled by the D1-V_t_ interaction^29^, whereas F-actin binding to V_t_ is controlled by a more complex mechanism involving interactions of D1 and D4 with both sides of V_t_^15,18,37^. The identification of several intermediate open conformations of vinculin suggests that the level of activation of vinculin is controlled by the number of auto-inhibitory contacts removed^42^. Our vinculin mutagenesis strategy confirmed that V_t_ activities are controlled by different combinations of V_h_-V_t_ autoinhibitory contacts. We show that the disruption of D1-V_t_ is not sufficient to expose the capping activity of V_t_, which requires the combined disruption of D1 and D4 contacts with V_t_, as previously suggested^18^. Early studies showed that the coincidental binding of talin and F-actin to D1 and V_t_ respectively keep vinculin in its open conformation^31^. In agreement with this model, full-length vinculin dissociates rapidly from FAs after actomyosin inhibition, whereas V_h_ remains stably associated, demonstrating that tensile actomyosin stress fibers bound to V_t_ maintain the open conformation of talin-bound vinculin^32,33^. This observation is further explained by the discovery that V_t_ forms a catch bond with F-actin in response to force^43^. It is therefore not surprising that the release of D1 and D4 from V_t_ in our experiments releases the capping activity of vinculin that occurs in the presence of actin filaments, but results in very little actin nucleation initiated without actin filaments.

Solving the problem of releasing the auto-inhibitory contacts of talin and vinculin was a prerequisite for determining the activity that results from the combination of their ABDs. It is interesting to remember that the combination of ABPs within transient complexes is often found in important signal-responsive mechanisms for regulating actin dynamics. However, the results of such combinations cannot be predicted from the activities of the isolated proteins, as exemplified by the formation of branched actin filaments resulting from the combination of WASP-related proteins and Arp2/3^4^. Here, we show that the combination of talin ABDs with vinculin V_t_ in a complex creates a machinery that nucleates actin filaments transiently capped at their barbed end (Fig. 6A). In this mechanism, talin and vinculin cooperate to form a new filament through a mechanism that is not yet understood but which probably involves the stabilization of actin nuclei, that form spontaneously in solution, by the ABDs of talin and vinculin. This mechanism leads to the transient barbed-end capping of the newly formed actin filaments. After being released from capping, the actin filament elongates by its free barbed end, while the complex remains attached to the side of the filament, near the pointed end (Fig. 6). It would be tempting to attribute to vinculin alone the nucleation and capping activities of the talin-vinculin complex, since V_t_ has these activities^18,44^. The fact that a complex, composed of active vinculin and talin with exposed VBSs but masked ABD1, does not nucleate, shows unambiguously the importance of ABD1 for nucleation. Although the function of the ABD1 domain of talin is generally restricted to integrin activation in a membrane-bound conformation that does not allow its binding to actin, it is important to recall here that talin is in equilibrium between actin and the plasma membrane as evidenced by its retrograde movement in FAs^7^. ABD2 is not involved in capping or nucleation mechanisms as demonstrated by the strong activities of a mutant that does not contain this domain. However, ABD3 can also combine with V_t_ to nucleate, suggesting a possible redundancy of ABD1 and ABD3 in this mechanism. It is interesting to note that the three ABDs of talin are redundant for the bundling of actin filaments^45^, but are involved more selectively in the actin polymerization activities of talin in combination with vinculin. It is very likely that actin filaments nucleated by talin and vinculin will end up forming large bundles, but our microscopy experiments, where the filaments are short and spaced out, disadvantages this activity and we have not explored this direction already widely documented by recent studies^45,46^.

The ability of a newly discovered nucleation machinery to form actin filaments in the presence of profilin has always been the subject of debate. Profilin prevents spontaneous polymerization of actin filaments in the cytoplasm where they would not play a role in producing force against the plasma membrane^4^. The generally accepted idea is that nucleation systems, located at the membrane and under the control of specific signaling pathways, trigger the polymerization of actin complexed with profilin. However, few nucleators are capable of nucleating actin filaments using profilin-actin complexes alone. Thus, the nucleation of branched filaments by the Arp2/3 complex or the nucleation of linear filaments by formins are severely inhibited in the presence of high profilin concentrations^47,48^, as observed for the talin-vinculin complex. It is likely that a pool of free actin, resulting from the constant depolymerization of actin networks, is transiently available to feed nucleators, such as the talin-vinculin machinery that we described, before it associates to profilin. In the cell, the concentration of profilin is lower than that of actin^49^. Actin is therefore partially free, or bound to other proteins, such as thymosin ß4, which sequesters actin monomers, or to other proteins that could be compatible with nucleators.

In the context of a stress fiber anchored to a focal adhesion containing talin and vinculin, the release of actin filament barbed ends by talin-vinculin complexes, occurring with a half-time around 100 s, may contribute to the slow elongation of a stress fiber. This release could also simply reinforce stress fibers by feeding them with new actin filaments. It would be interesting to determine how the talin-vinculin complex cooperates with other ABPs in FAs, such as VASP, parvin, or tensin, to regulate actin assembly. The activities we have identified should generate filaments of different orientations in FAs. Actin filaments should be oriented with their barbed end facing the focal adhesion if the talin-vinculin complex caps their barbed end, or oriented with the barbed end towards the cell body or the leading edge if they have been nucleated and released. Although early electron microscopy studies concluded that the actin filaments making up the ends of stress fibers near the cell front are predominantly oriented with their barbed ends towards the membrane^50^, later studies benefiting from the precision of cryoelectron tomography have somehow revised these conclusions. These studies show that the stress fibers in contact with the front part of a focal adhesion contain filaments whose barbed ends are oriented towards the leading edge of the cell, and filaments of mixed polarities at the rear of the adhesion^51^, which is compatible with our findings (Fig. 6B). The same orientations have been observed in platelet pseudopodia^52^. The ability of the talin-vinculin complex to generate actin structures containing antiparallel filaments should promote contractility by myosin II.

## Methods

### Recombinant cDNA constructs

cDNAs encoding for vinculin 1-1066 (V_FL_), vinculin E28K/D33H (V_1a_), vinculin E28K/D33H/D110H/R113E/N773I/E775K (V_1ab4_) were synthesized and subcloned into the NcoI site of pET-3d by Genscript. pGEX-6P-1-T_F2F3R1R2R3_ was obtained by PCR amplification of the talin-1 cDNA with primers Ta-196-Bam-CLC (Supplementary Table 2) and 3-primer-911-EcorI (Supplementary Table 2) and cloning into the BamH1 and EcoRI sites of pGEX-6P-1 plasmid (Cytiva). The pETM T_R2R3ABD3_ plasmid was obtained by PCR amplification of the talin-1 R2R3 cDNA with the o217 and o218 primers (Supplementary Table 2), and the talin-1 R13 (ABD3) cDNA with the o220 and o221 primers (Supplementary Table 2), followed by BamHI ligation and cloning into the KpnI and EcoRI sites of a pETM-11 plasmid (Novagen) previously modified to include a cDNA encoding a N-terminal Strep-tagII followed by PreScission site (pETM-11sp). The pETM TΔ1ΔAIΔABD2 plasmid was obtained by PCR amplification of two talin-1 fragments obtained with the o174 and o224 primers (Supplementary Table 2), and the o225 and o228 primers (Supplementary Table 2), respectively, followed by their SmaI ligation and cloning into the NheI and NcoI sites of the pETM-11sp. Plasmids encoding for vinculin 879-1066 (V_t_), talin-1 482–636 (VBS_1_), talin-1 1655–1822 (R9), full-length talin-1 (T_FL_), EGFP-Vh were described previously^18,22,38^. cDNAs of TΔAI, TΔ1, TΔ2, TΔ3, TΔ1ΔAI, TΔ2ΔAI, TΔ3ΔAI were constructed and subcloned into pET-29a(+) by Genscript. The constructs were verified by sequencing. See Supplementary Fig. 4 for the detail of the talin constructs.

The pBFP-Talin _FL_ and pBFP-T_Δ1ΔAI_ plasmids were obtained through Gibson assembly of the pTagBFP-C1 backbone amplified by PCR with the primers Backbone-TagBFP-fwd and Backbone-TagBFP-rev (Supplementary Table 2), and the T_FL_ and T_Δ1ΔAI_ inserts amplified with the primers Insert-Talin-fwd and Insert-Talin-rev (Supplementary Table 2), using T_FL_ and T_Δ1ΔAI_ cDNAs as templates respectively. The pmCherry-Vinculin_FL_ and pmCherry-V_1ab4_ plasmids were obtained through Gibson assembly of the pmCherry backbone amplified by PCR with the primers Backbone-mCherry-fwd and Backbone-mCherry-rev (Supplementary Table 2), and the Vinculin_FL_ and V_1ab4_ inserts amplified with the primers Insert-Vinculin-fwd and Insert-Vinculin-rev (Supplementary Table 2), using vinculin_FL_ and V_1ab4_ cDNAs as templates respectively. The plasmid encoding EGFP-actin was a gift from B. Imhof (University of Geneva).

### Protein purification

V_FL_, EGFP-Vh, V_t_, V_1a_, V_1ab4_, T_FL_, TΔAI, TΔ1, TΔ2, TΔ3, TΔ1ΔAI, TΔ2ΔAI, TΔ3ΔAI, TΔ1ΔAIΔABD2, T_F2F3R1R2R3_, T_R2R3ABD3_ and VBS_1_ were expressed in *E.Coli* BL21 as previously described^38^. Briefly, 1 mM isopropyl β-D-1-thiogalactopyranoside (IPTG) was used for induction. Bacterial pellets of V_FL_, V_t_, V_1a_, V_1ab4_ were lysed by sonication in 20 mM Tris, pH 8.0, 1 M NaCl, 1 mM β-mercaptoethanol, 10 μg/ml benzamidine and 1 mM PMSF. Lysates of His-tagged vinculin constructs were purified by Ni-NTA-sepharose affinity chromatography (Ni^2+^-nitrilotriacetic acid, Qiagen), followed by a Q-Sepharose ion exchange column. V_t_ was purified as previously described^18^. Vinculin proteins were finally dialyzed in 20 mM Tris, pH 7.8, 1 mM DTT. Bacterial pellets of T_FL_, TΔAI, TΔ1, TΔ2, TΔ3, TΔ1ΔAI, TΔ2ΔAI, TΔ3ΔAI, TΔ1ΔAIΔABD2, T_F2F3R1R2R3_, T_R2R3ABD3_ were lysed by sonication in 50 mM Tris, pH 7.8, 500 mM NaCl, 1% Triton X-100, 1 mM β-mercaptoethanol, 10 μg/ml benzamidine and 1 mM PMSF. Lysates of His-tagged talin constructs were purified by Ni-NTA-sepharose affinity chromatography (Ni^2+^-nitrilotriacetic acid, Qiagen), followed by a gel filtration column (Superdex 200, 16/600, GE Healthcare). VBS_1_ was purified as previously described^18^. Talin constructs were finally dialyzed in 20 mM Tris, pH 7.8, 100 mM KCl, 1 mM DTT.

### F-actin co-sedimentation assay

F-actin co-sedimentation assays were performed to determine the affinity of vinculin constructs for F-actin in the absence and presence of talin VBS_1_. In F-actin co-sedimentation assay with vinculin alone, 2 μM of vinculin (V_FL_, V_t_, V_1a_, V_1ab4_) was incubated with 0, 1.5, 3, 4.5, 6, and 10 μM F-actin in 5 mM Tris, pH 7.8, 100 mM KCl, 1 mM MgCl_2_, 0.2 mM EGTA, 200 μM ATP, 1 mM DTT during 15 min at room temperature. To test the effect of VBS_1_, F-actin co-sedimentation assays were also performed in the presence of 2 μM of vinculin (V_FL_, V_1a_), 10 μM F-actin, with or without 1, 3, 5, 10 μM VBS_1_ in 5 mM Tris, pH 7.8, 100 mM KCl, 1 mM MgCl_2_, 0.2 mM EGTA, 200 μM ATP, 1 mM DTT during 15 min at room temperature. After centrifugation at 300,000 x g in a TLA-120.1 rotor (Beckman) during 30 min, the pellets and supernatants were separated and loaded on SDS-PAGE. Gels were scanned and analyzed with the ImageJ software.

### Polymerization assay

Actin polymerization was measured by the increase in fluorescence of 10 % pyrenyl-labeled actin in a SAFAS Xenius spectrofluorimeter (Safas, Monaco). To measure the elongation of actin filament barbed ends, actin polymerization was induced by adding 100 pM spectrin-actin seeds to 10% pyrenyl-labeled CaATP-G-actin in 5 mM Tris, pH 7.8, 100 mM KCl, 1 mM MgCl_2_, 0.2 mM EGTA, 200 μM ATP, 1 mM DTT in presence of indicated proteins. The fraction of barbed end elongation was calculated as the ratio between the elongation rates in the presence and absence of proteins of interest. To test the ability of proteins to nucleate actin filaments, spontaneous polymerization was induced by adding 10% pyrenyl-labeled CaATP-G-actin in 5 mM Tris, pH 7.8, 25 mM KCl, 1 mM MgCl_2_, 0.2 mM EGTA, 200 μM ATP, 1 mM DTT in presence of the proteins of interest or in 5 mM Tris, pH 7.0, 100 mM KCl, 1 mM MgCl_2_, 0.2 mM EGTA, 200 μM ATP, 1 mM DTT at 37 °C.

### Observation of talin, vinculin, and single actin filaments in TIRF microscopy

Our protocol is a modification of protocols used to study talin and vinculin activities^18,22^. To prevent the nonspecific binding of actin filaments to the surface of the coverslip, we first irradiated coverslips with deep UVs for 3 min and incubated them with 0.1 mg/mL PLL-PEG for 1 h at room temperature. The coverslip was then washed extensively with water and dried. Flow cells containing 40-60 μl of liquid were prepared by sticking the PLL-PEG-coated coverslip to a slide with double-sided adhesive spacers. In experiments to observe and count single filament number, the chamber was first incubated with washing buffer (5 mM Tris, pH 7.8, 200 μM ATP, 1 mM DTT, 1 mM MgCl_2_, 0.2 mM CaCl_2_, 25 mM KCl) for 1 min. The chamber was then saturated with 10% BSA for 5 min and washed with washing buffer. The final reaction was then injected into the chamber. A typical reaction was composed of 1 μM actin (5% Alexa488-labeleld) in 5 mM Tris, pH 7.8, 200 μM ATP, 1% methylcellulose, 5 mM 1,4-diazabicyclo(2,2,2)-octane (DABCO), 25 or 100 mM KCl, 1 mM MgCl_2_, 200 μM EGTA, 40 mM DTT supplemented with various talin and vinculin mutants. To observe talin, vinculin and actin simultaneously, 0.2 µM TΔ1ΔAI (78% Alexa594-labeled) and 1 µM V_1ab4_ (18% Alexa647-labeled) were first mixed, diluted 20 times to reach final concentrations of 10 nM TΔ1ΔAI and 50 nM V_1ab4_, injected on a flow chamber to immobilize TΔ1ΔAI-V_1ab4_ complexes non-specifically on the surface passivated with PLL-PEG, and finally supplemented with 0.8 µM actin (5% Alexa488-labeled). Finally, we sealed the flow chamber with VALAP (a 1:1:1 mixture of vaseline, lanolin, and paraffin) and observed the reaction on a Nikon Eclipse Ti-E inverted microscope equipped with 60X/1.49NA objective (Nikon). The time-lapse videos were acquired by Metamorph and subsequently analyzed by the ImageJ software.

### Micropatterning

Coverslips were first cleaned by successive sonication in water and ethanol before being dried and then irradiated for 1 min under a UV lamp at 160 nm (Ossila). They were then incubated with a 0.1 mg/ml solution of PLL-PEG (SuSoS) for 2 h at room temperature. The coverslips were then placed with a drop of water on the washed and UV-activated chromium-quartz photomask, irradiated for 4 min, recovered and dried. Flow cells were prepared by sticking the PLL-PEG-coated coverslip to a slide with double-sided adhesive spacers. After addition of the proteins, the chamber was sealed with VALAP (a mixture of vaselin, lanolin and paraffin).

### Protein labeling

For 3-color imaging, actin was labeled with Alexa Fluor 488 carboxylic acid, succinimidyl ester, which reacts with the NH_2_ groups of basic amino acids, as previously described^38^. TΔ1ΔAI and V_1ab4_ were labeled on free cysteines with Alexa Fluor 594 maleimide and Alexa Fluor 647 maleimide respectively. Proteins were dialysed in 20 mM Tris HCl pH 7.8 and 100 mM KCl (TΔ1ΔAI) or 20 mM Tris HCl pH 7.8 (V_1ab4_) overnight at 4 °C, then incubated for 3 hours with a 10-fold excess of Alexa Fluor 594 maleimide (TΔ1ΔAI) or Alexa Fluor 647 maleimide (V_1ab4_). The reaction was then stopped with 1 mM DTT and dialysis was performed in 20 mM Tris HCl pH 7.8, 100 mM KCl and 1 mM DTT (TΔ1ΔAI) or 20 mM Tris HCl pH 7.8, 1 mM DTT (V_1ab4_), overnight at 4 °C and protected from light. Finally, the labeling ratio was calculated using the UV-visible absorbance spectrum and the molar extinction coefficients of the proteins and the dyes. The protein was then frozen in liquid nitrogen and stored at −80 °C.

### Cell lines and transfection

Hela cells (ATCC number CCL-2) were maintained in DMEM medium (Life Technologies) supplemented with 10% fetal bovine serum (FBS) (Biochrome) and 100 units/ml penicillin-streptomycin (anprotec) at 37 °C with 5% CO_2_. 24 h before transfection, 300,000/well Hela cells were seeded in a 6-well plate. Hela cells were transfected using Fugene (Promega) and incubated for 48 h with pmCherry-Vinculin _FL_ / pBFP-Talin _FL_ or pmCherry-V_1ab4_ / pBFP-T_Δ1ΔAI_ to be observed after fixation or, after additional transfection of pEGFP-actin, submitted to FRAP.

### Observation of fixed cells

Coverslips were incubated with 1 μg/cm^2^ fibronectin (Merck) for 45 min. Transfected cells were seeded to fibronectin-coated coverslips. After 6 h, Hela cells were fixed in 4% paraformaldehyde in PBS (Santa Cruz) for 15 min at 37 °C and permeabilized for 10 min with 0.3% Triton X-100 (Thermofisher) in PBS. Fixed cells were blocked for 1 h with 5% FBS in PBS at 4 °C followed by 1 h staining with Alexa488-phalloidin (Thermofisher) and mounted in mounting media (Thermofisher). Images were acquired on an LSM 800 confocal laser scanning microscope (Zeiss) equipped with a 63×/1.4 NA oil objective with Airyscan.

### FRAP experiments

The FRAP experiment was performed with an LSM 800 confocal laser scanning microscope (Zeiss) equipped with a 63×/1.4 NA oil objective with Airyscan at 37 °C with 5% CO_2_. EGFP-actin was bleached by a 10 times scan on the selected region with 10% intensity of 488 nm laser (10 mW). After bleaching, time-lapse images were acquired every 5 s. Images were analyzed by the Image J software (NIH).

### Statistics and reproducibility

Statistical analyses and graphical representations were carried out using GraphPad/Prism, Kaleidagraph, or Excel. Information on exact n values, statistical tests and their description, and error bars are given in the figure legends and the Source data file. No statistical method was used to predetermine sample size. No data were excluded from the analyses. The experiments were not randomized. The reproducibility is indicated in the figure legends.

### Reporting summary

Further information on research design is available in the Nature Portfolio Reporting Summary linked to this article.

## Supplementary information

Supplementary Information

Reporting Summary

Description of Additional Supplementary Files

Supplementary Movie 1

Supplementary Movie 2

Supplementary Movie 3

Supplementary Movie 4

Supplementary Movie 5

Supplementary Movie 6

Supplementary Movie 7

Supplementary Movie 8

Supplementary Movie 9

Transparent Peer Review file

## Source data

Source data file

## Data Availability

Our manuscript is accompanied by a Source Data file providing the data used in Figs. 1B–E, 2B–E, 3E–H, 4E, and 5C, D, and Supplementary Figs. 2A–E, 3A–C, 6C–E, 7A–D, 8A–D, 9A–E, 10A–D, 11A–E, 12A–C, 13A–E, 14A–C, 15A–D, 16A–F, 17A, B, 18A, B, 19A–C, 20A, B, 21A, B, and 22C, D. Source data are provided with this paper.
