# Supplementary material for: Talin and vinculin combine their activities to trigger actin assembly
Source: Nat Commun. 2024 Nov 3;15:9497. doi: 10.1038/s41467-024-53859-1 (PMC11532549; doi:10.1038/s41467-024-53859-1)
Supplement: Supplementary file 1 — Supplementary Information [file 41467_2024_53859_MOESM1_ESM.pdf]

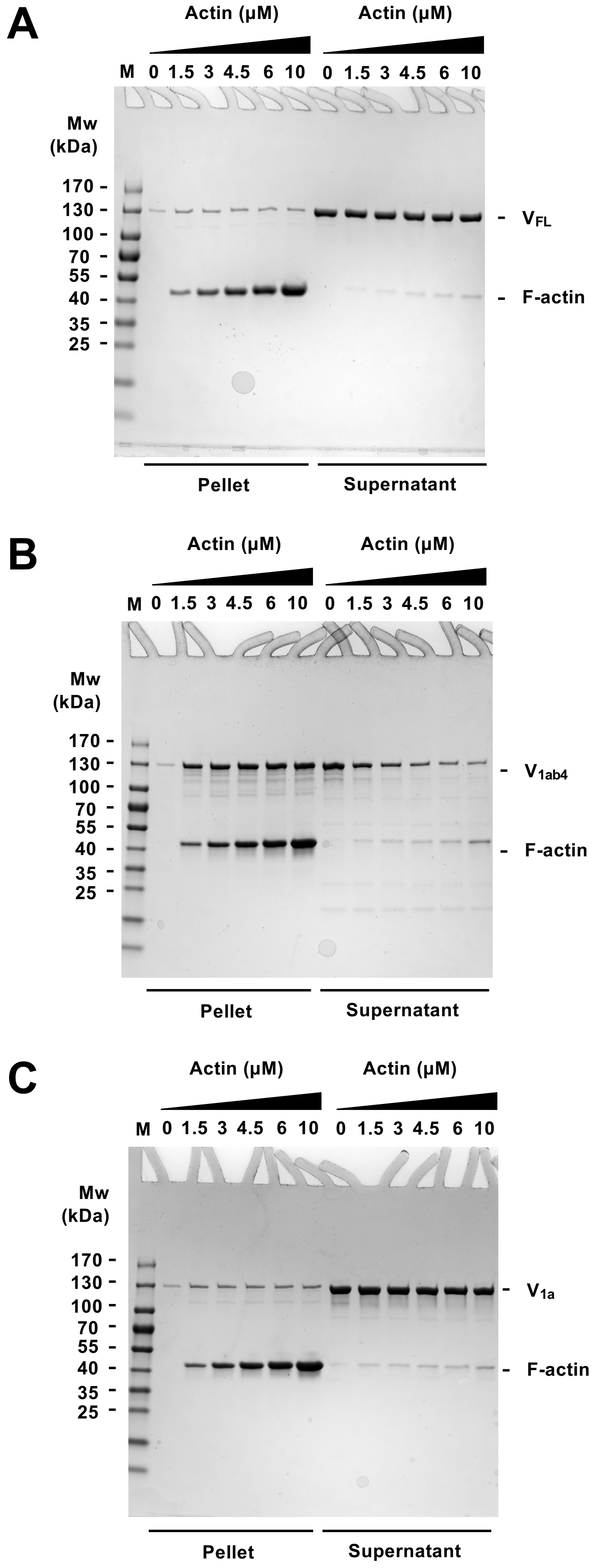

Supplementary figure 1. The high affinity binding of vinculin to the side of actin filaments requires the disruption of the contacts made by V_t with both D1 and D4. (A-C) SDS-PAGE gels showing the supernatant and pellet fractions of cosedimentation assays containing 2 μM of the indicated vinculin mutants and increasing concentrations of F-actin (0, 1.5, 3, 4.5, 6, 10 μM). The gels show on the left a lane labelled M which corresponds to a molecular weight scale (Mw) whose values are given in kDa (kilodalton). These experiments were repeated three times independently with the same results.

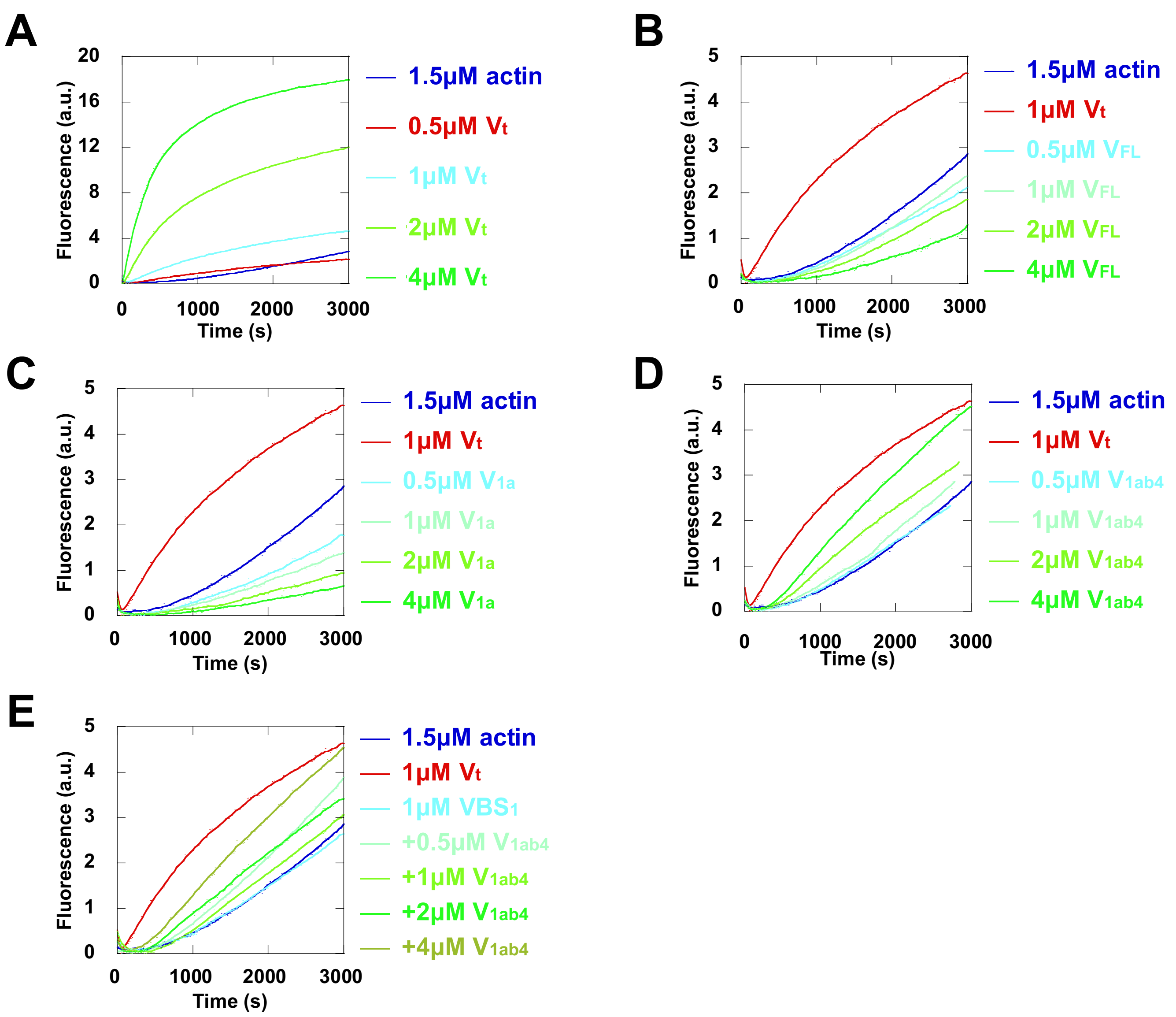

Supplementary figure 2. Talin VBS$_1$ combined with D4-V$_t$ release has no effect on nucleation by vinculin. (A-D) Spontaneous actin polymerization was measured in the presence of increasing concentrations of the indicated vinculin mutants and 1.5 µM actin (10% pyrenyl-labeled) in a low salt buffer (25 mM KCl) in the absence of VBS$_1$ **(A-D)** and in presence of 1 µM VBS$_1$ **(E)**. The control kinetics showing the polymerization of 1.5 µM actin alone, as a negative control, and in the presence of 1 µM V$_t$, as a positive control, are the same in all panels. Fluorescence is expressed in arbitrary units (a.u.). Source data are provided as a Source Data file. **(A-E)** These experiments were repeated twice independently with the same results.

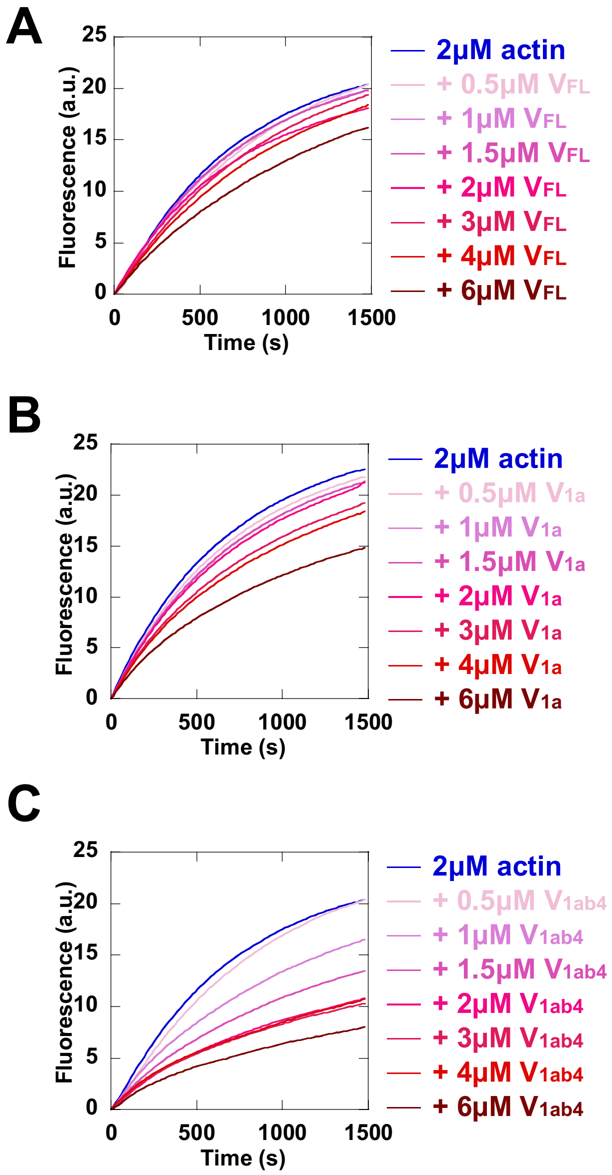

Supplementary figure 3. The release of the D1-V_t and D4-V_t contacts allows barbed-end capping by vinculin. (A-C) The elongation of actin filament barbed end was measured in the presence of increasing concentrations of the indicated vinculin mutants, 100 pM spectrin-actin seeds, 2 µM actin (10% pyrenyl-labeled). The control kinetics showing the polymerization of 2 µM actin alone are the same for the panels A and C performed the same day, while a different control curve is used for B performed on a different day. Fluorescence is expressed in arbitrary units (a.u.). Source data are provided as a Source Data file. **(A-C)** These experiments were repeated twice independently with the same results.

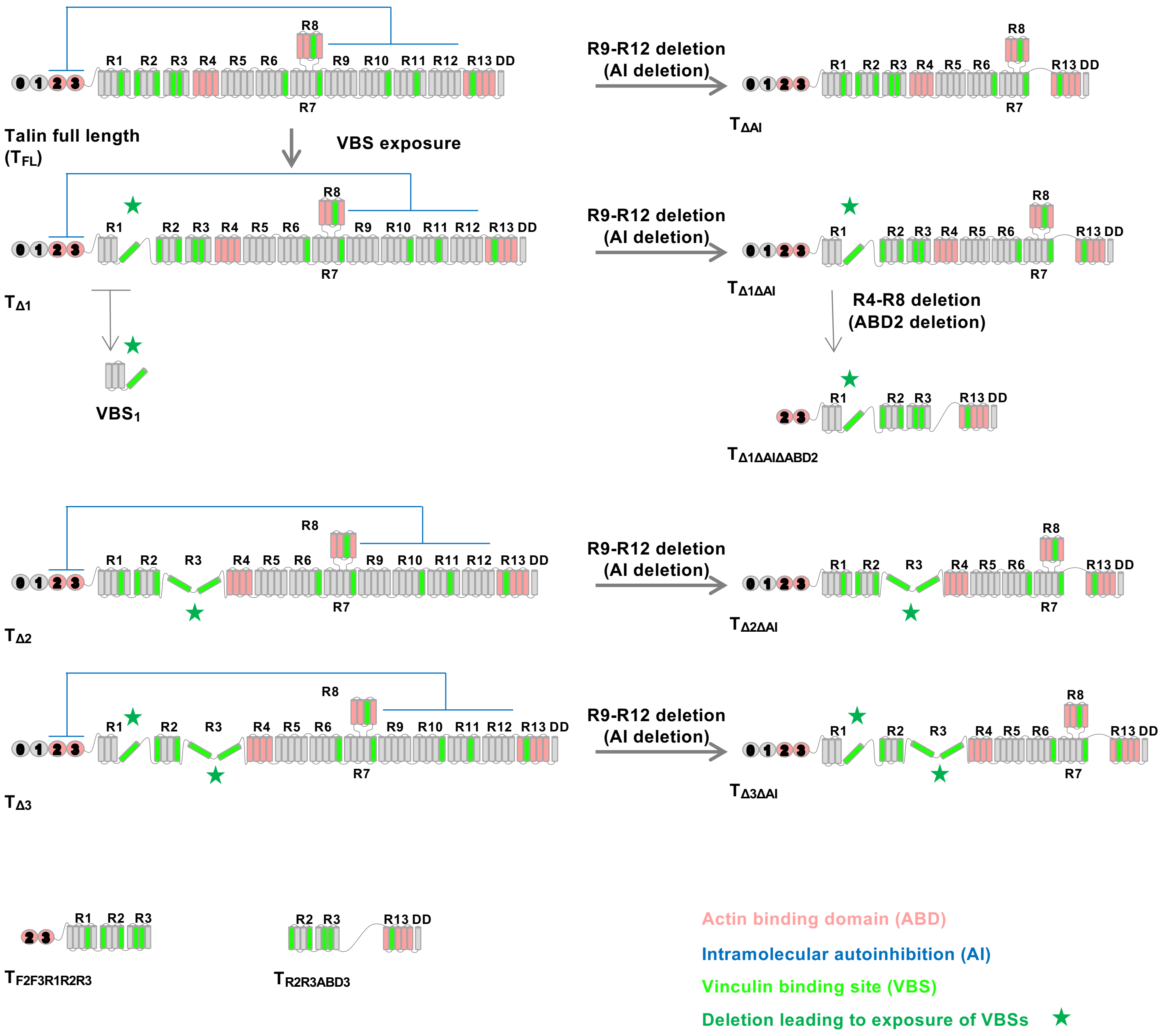

Supplementary figure 4. Talin constructs used in the study. Domain organisation of full-length talin and talin constructs featuring intramolecular autoinhibitions in blue, VBSs in green and ABDs in pink. Grey bars indicate α-helices of the rod. Green stars indicate exposed VBSs.

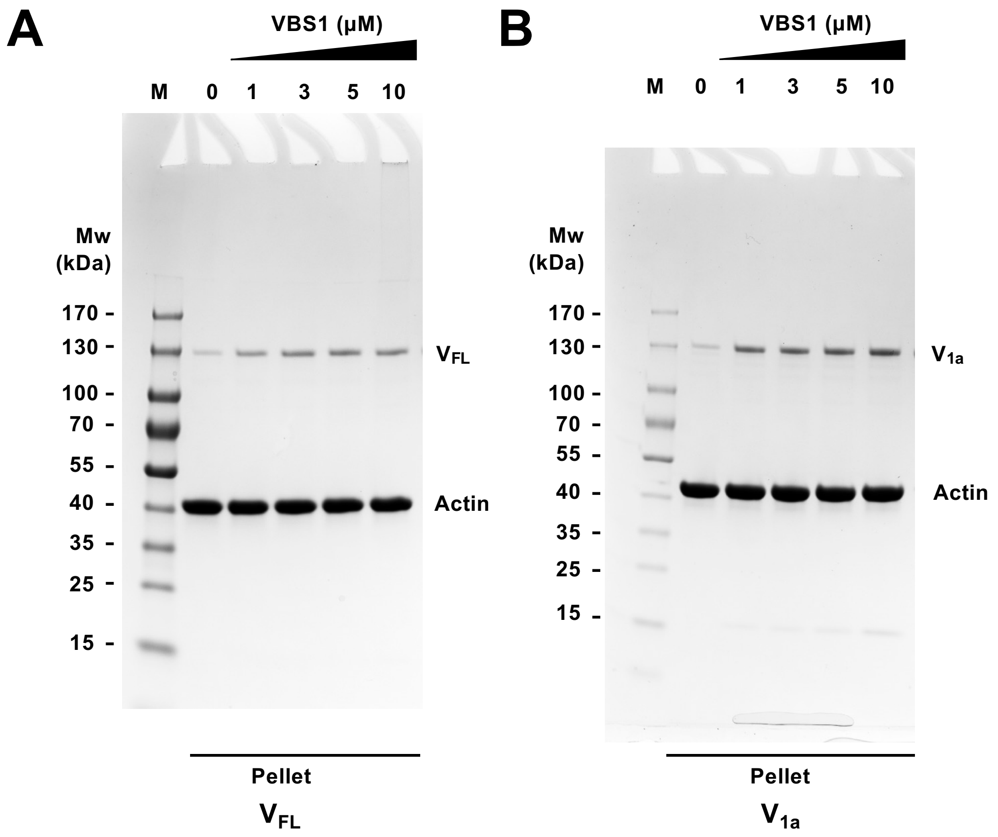

Supplementary figure 5. Talin VBS_1 combined with D4-V_t release induces vinculin binding to F-actin. (A, B) SDS-PAGE gels showing the pellet fractions of F-actin co-sedimentation reactions containing 2 µM of the indicated vinculin mutants, 10 µM F-actin and increasing concentrations of talin VBS_1 (0, 1, 3, 5, 10 µM). The gels show on the left a lane labelled M which corresponds to a molecular weight scale (Mw) whose values are given in kDa (kilodalton). These experiments were repeated three times independently with the same results.

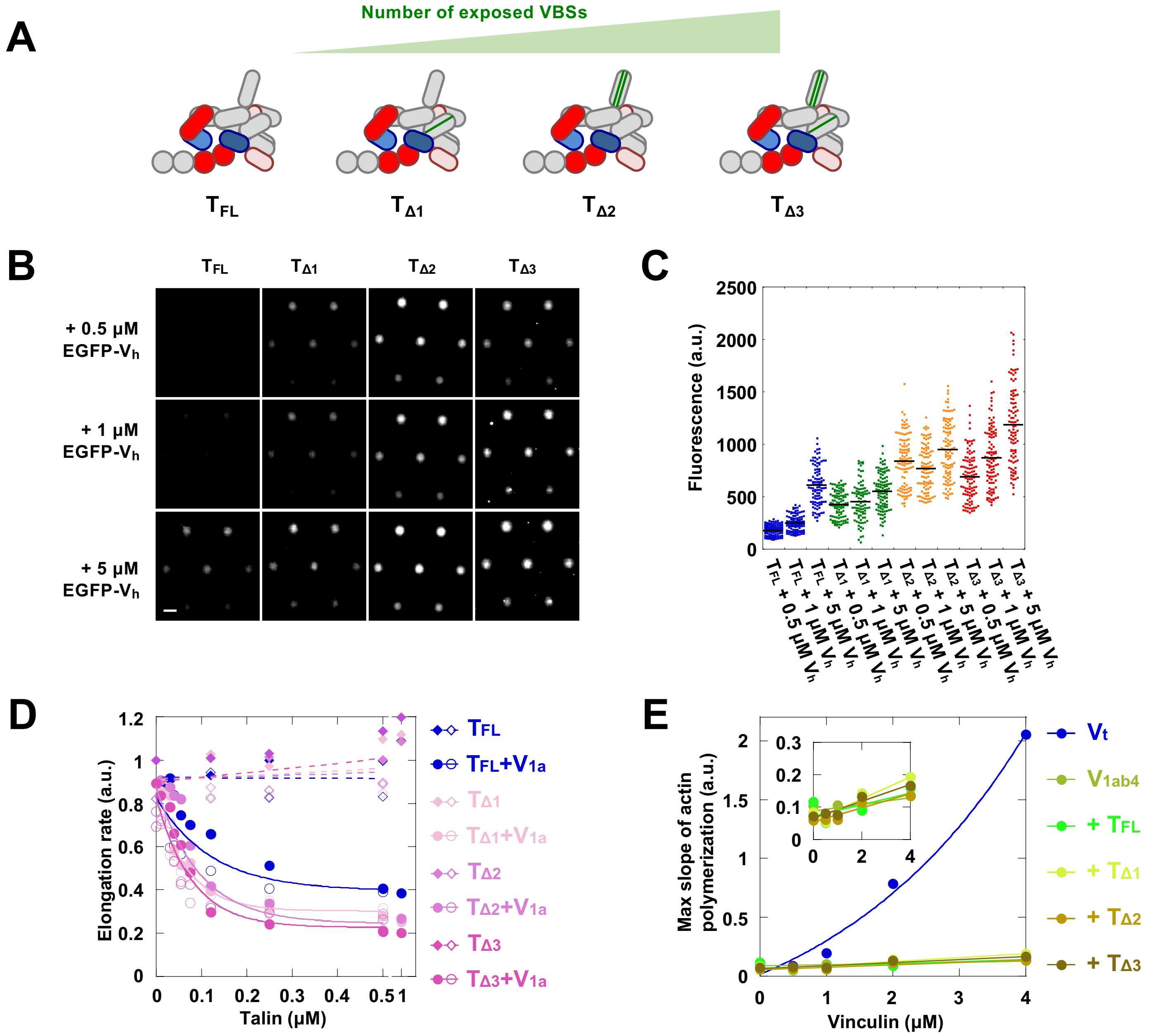

Supplementary figure 6. Full-length talin with exposed VBSs promotes vinculin-dependent barbed-end capping but not nucleation. (A) Schematic representation of talin constructs with increasing numbers of exposed VBS used in (B-E). **(B, C)** Exposure of VBSs in talin increases the binding of vinculin. **(B)** A micropatterned surface is incubated with 1 µM of the indicated talin contructs, washed, incubated with 0.5 µM, 1 µM and 5 µM EGFP-V_h and imaged in TIRF microscopy. Scale bar = 10 µm. **(C)** Quantification of the experiment presented in (A). Each data point represents the mean fluorescence of EGFP-V_h in one disk. The bar shows the mean. **(D)** Actin filament barbed end elongation is measured in the presence of spectrin-actin seeds (100 pM), actin (1.5 µM, 10% pyrenyl-labeled) and increasing concentration of talin mutants, in the absence and presence of vinculin V_{1a} (2 µM) (Supplementary Figure 7 and Supplementary Figure 8). The fraction of barbed end elongation was calculated as the ratio of the elongation rate in the presence of increasing concentrations of talin, in the presence or absence of V_{1a}, to the elongation rate of actin alone. Note the break between 0.5 µM and 1 µM on the x-axis. Open and closed symbols indicate two independent experiments in the same conditions. **(E)** The maximal rate of spontaneous actin polymerization (1.5 µM, 10% pyrenyl-labeled) is plotted against increasing concentrations of V_{1ab4} alone and in the presence of 1 µM of the indicated talin mutants (Supplementary Figure 10). V_t alone is used as a positive control for nucleation. The inset is a zoom without V_t. This experiment was repeated twice independently with the same results. **(C-E)** Fluorescence and actin polymerization are expressed in arbitrary units (a.u.). **(C-E)** Source data are provided as a Source Data file.

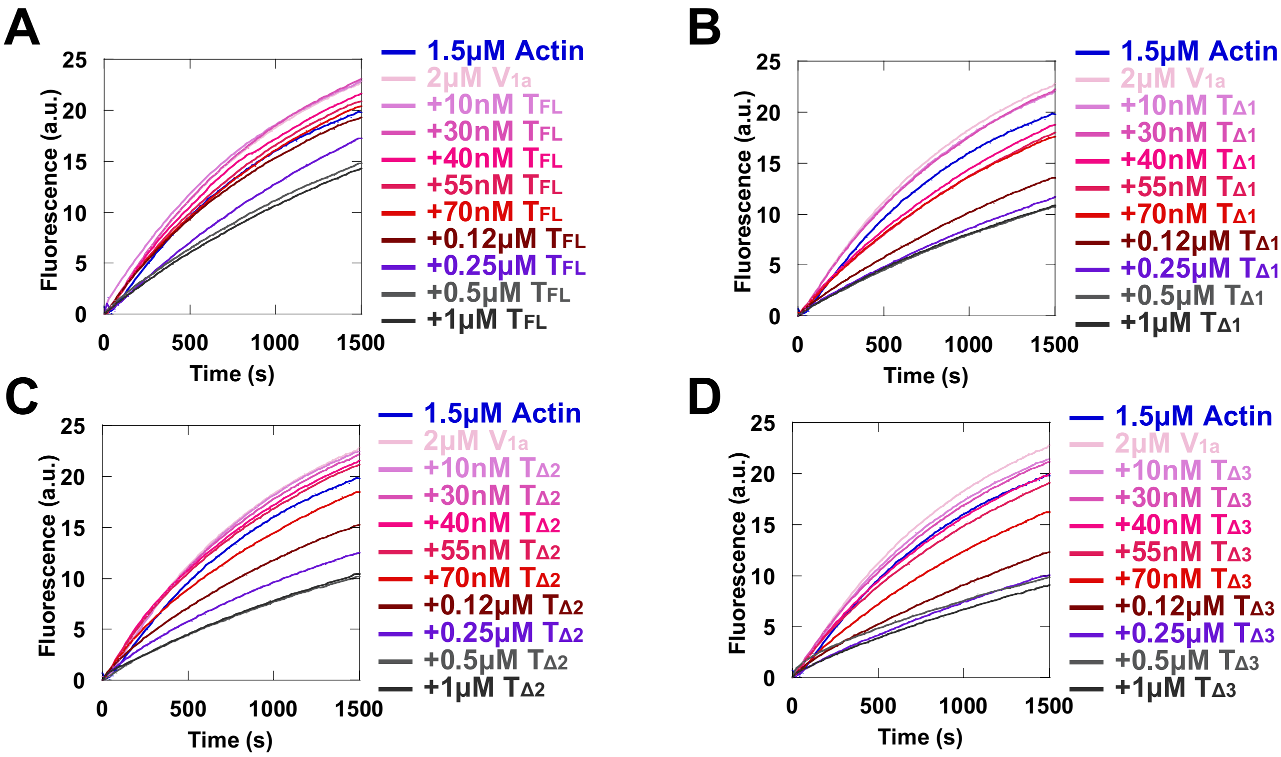

Supplementary figure 7. A complex composed of full-length talin with exposed VBSs and V_{1a} caps actin filament barbed ends. (A-D) The elongation of actin filament barbed end was measured in the presence of 2 µM V_{1a} with increasing concentrations of the indicated talin mutants, 100 pM spectrin-actin seeds and 1.5 µM actin (10% pyrenyl-labeled). The control kinetics showing the polymerization of 1.5 µM actin in the presence of 2 µM V_{1a} are the same in all panels. Fluorescence is expressed in arbitrary units (a.u.). Source data are provided as a Source Data file. **(A-D)** These experiments were repeated twice independently with the same results.

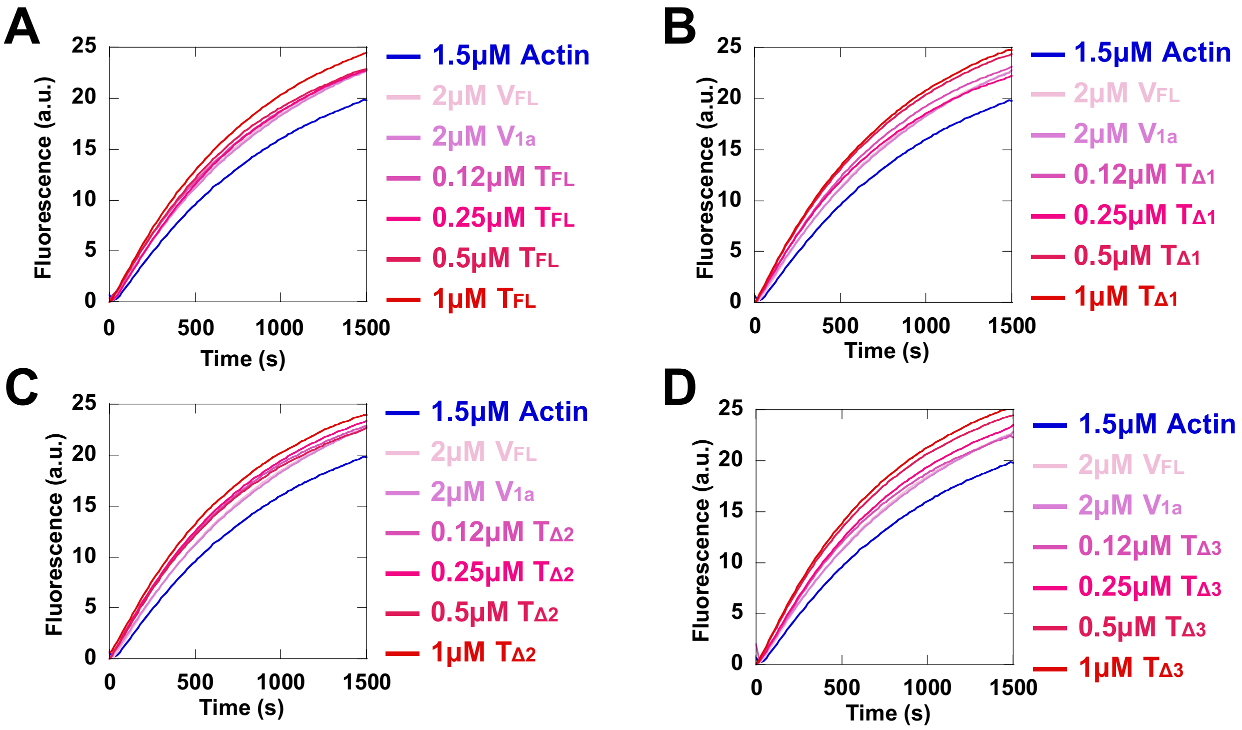

Supplementary figure 8. Talin mutants with exposed VBSs alone do not cap barbed ends. (A-D) The elongation of actin filament barbed end was measured in the presence of increasing concentrations of the indicated talin mutants, 100 pM spectrin-actin seeds, 1.5 µM actin (10% pyrenyl-labeled). The kinetics with 1.5 µM G-actin, 2 µM V_{FL} or 2 µM V_{1a}, used here as additional negative controls, are the same in all panels. Fluorescence is expressed in arbitrary units (a.u.). Source data are provided as a Source Data file. **(A-D)** These experiments were repeated twice independently with the same results.

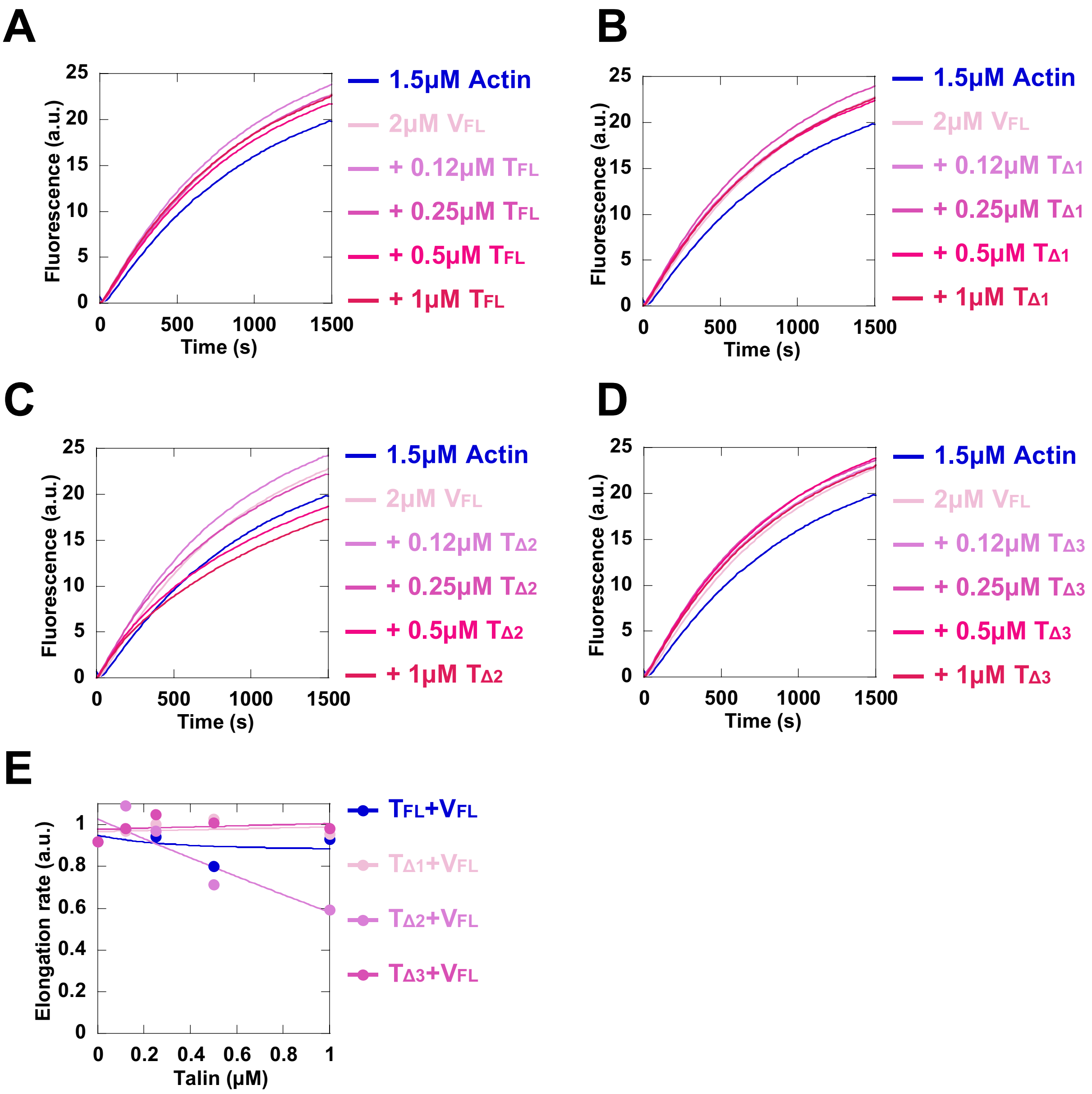

Supplementary figure 9. Addition of full-length talin with exposed VBSs to V_{FL} does not cap actin filament barbed ends. (A-D) The elongation of actin filament barbed end was measured in the presence of 2 µM V_{FL}, increasing concentrations of the indicated talin mutants, 100 pM spectrin-actin seeds, 1.5 µM actin (10% pyrenyl-labeled). The kinetics with 1.5 µM actin alone and with 2 µM V_{FL} are the same in all panels. **(E)** The fraction of barbed end elongation was calculated as the ratio of the elongation rate in the presence of V_{FL} and talin mutants to the elongation rate of 1.5 µM actin alone. **(A-E)** Fluorescence and elongation rates are expressed in arbitrary units (a.u.). Source data are provided as a Source Data file.

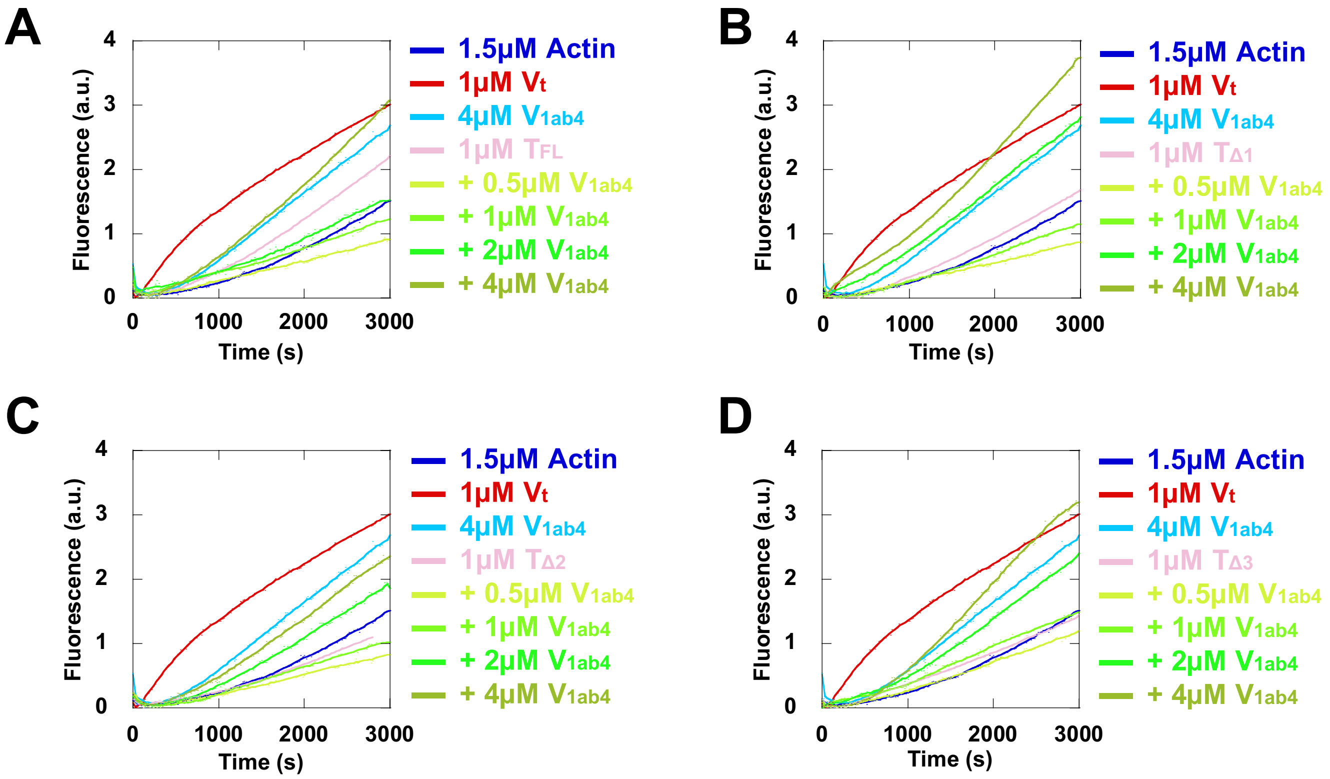

Supplementary figure 10. Talin mutants with exposed VBSs combined with V_{1ab4} do not stimulate actin assembly. (A-D) Spontaneous actin polymerization was measured in the presence of 1 μM of the indicated talin mutants, increasing concentrations of V_{1ab4} and 1.5 μM actin (10% pyrenyl-labeled) in a low salt buffer (25 mM KCl). The kinetics with 1.5 μM actin alone, 1.5 μM actin and 1 μM V_t used as a positive control for nucleation, and 1.5 μM actin and 4 μM V_{1ab4} are the same in all panels. Fluorescence is expressed in arbitrary units (a.u.). Source data are provided as a Source Data file. **(A-D)** These experiments were repeated twice independently with the same results.

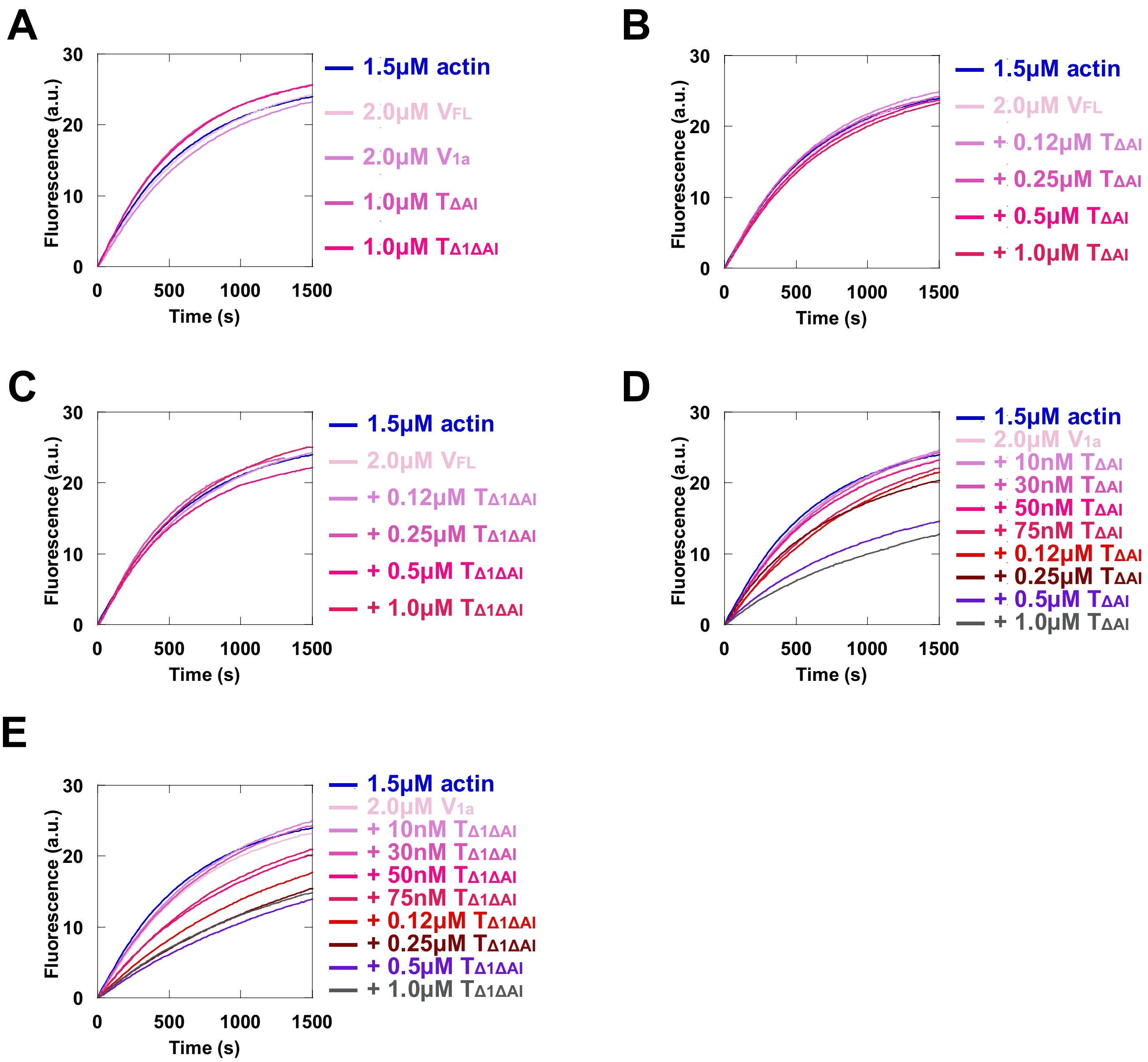

Supplementary figure 11. Talin mutants, with released autoinhibitory contacts and one exposed VBS combine with V_{1a} to cap actin filament barbed ends. The elongation of actin filament barbed end was measured in the absence and presence of 2 µM V_{FL} or 2 µM V_{1a} or 1 µM $T_{\Delta AI}$ or 1 µM $T_{\Delta 1\Delta AI}$ **(A)**, in the presence of increasing concentrations of indicated talin mutants and 2 µM V_{FL} **(B-C)** or 2 µM V_{1a} **(D-E)**, 100 pM spectrin-actin seeds and 1.5 µM actin (10% pyrenyl-labeled). The control kinetics with 1.5 µM actin and 2 µM V_{FL} **(A-C)** or 1.5 µM actin and 2 µM V_{1a} **(D-E)** are the same in all panels. Fluorescence is expressed in arbitrary units (a.u.). Source data are provided as a Source Data file. **(A-E)** These experiments were repeated twice independently with the same results.

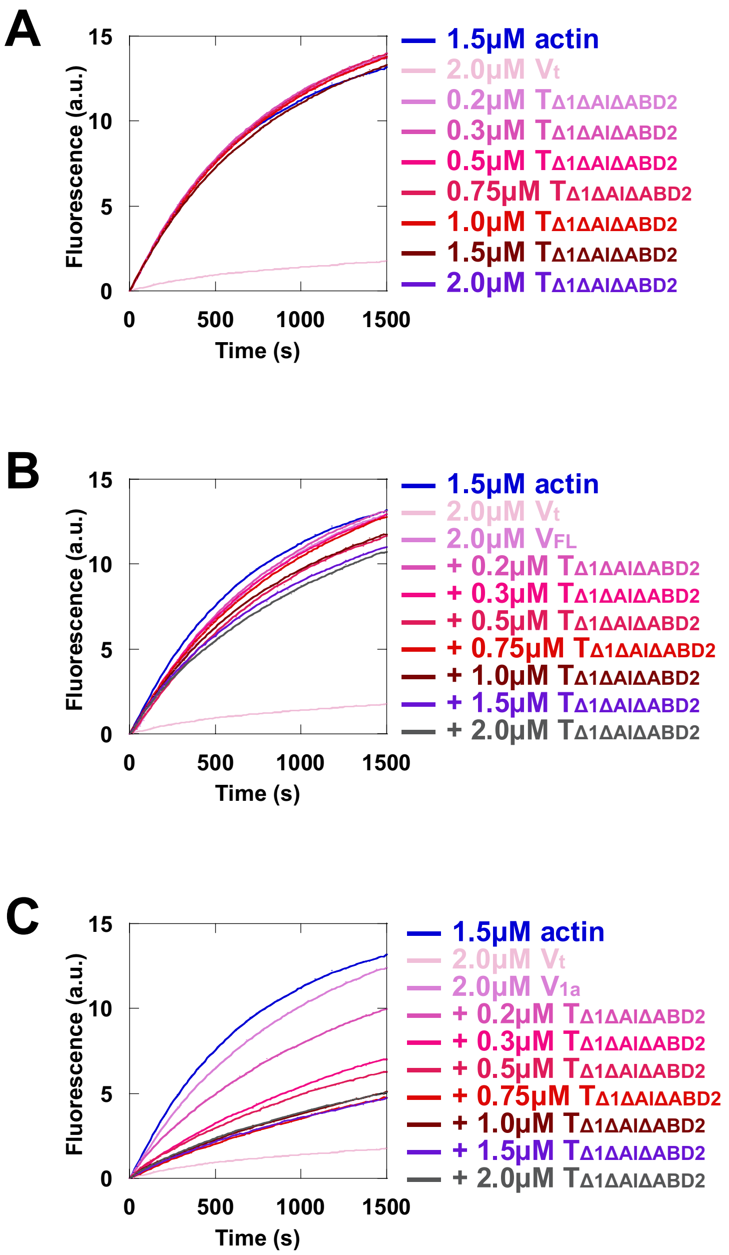

Supplementary figure 12. A talin mutant, with released autoinhibitory contacts, one exposed VBS and lacking ABD2, combines efficiently with V_{1a}, but not with V_{FL}, to cap actin filament barbed ends. (A-C) The elongation of actin filament barbed end was measured in the presence of increasing concentrations of $T_{\Delta1\Delta AI\Delta ABD2}$, 100 pM spectrin-actin seeds and 1.5 µM actin (10% pyrenyl-labeled) **(A)**, increasing concentrations of $T_{\Delta1\Delta AI\Delta ABD2}$, 2 µM V_{FL} **(B)** or 2 µM V_{1a} **(C)**, 100 pM spectrin-actin seeds and 1.5 µM actin (10% pyrenyl-labeled). The kinetics with 1.5 µM actin alone and 1.5 µM actin and 2 µM V_t are the same in all panels. Fluorescence is expressed in arbitrary units (a.u.). Source data are provided as a Source Data file. **(A-C)** These experiments were repeated twice independently with the same results.

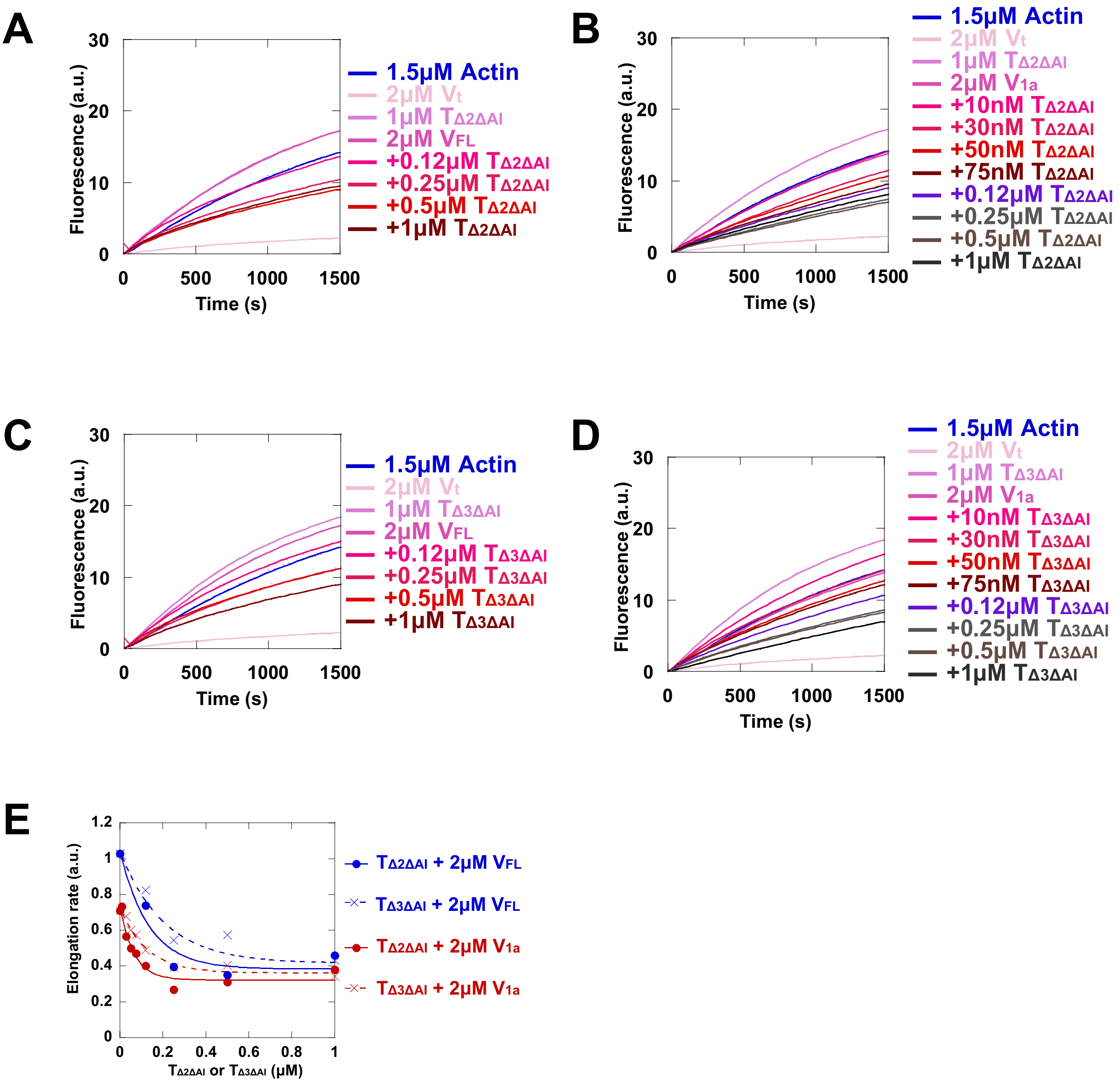

Supplementary figure 13. Talin mutants, with released autoinhibitory contacts and two or three exposed VBSs, combine with V_{FL} and V_{1a} to cap actin filament barbed ends. (A-E) The elongation of actin filament barbed end was measured in the presence of increasing concentrations of $T_{\Delta 2\Delta AI}$ and 2 μM of V_{FL} **(A)**, increasing concentrations of $T_{\Delta 2\Delta AI}$ and 2 μM of V_{1a} **(B)**, increasing concentrations of $T_{\Delta 3\Delta AI}$ and 2 μM of V_{FL} **(C)**, increasing concentrations of $T_{\Delta 3\Delta AI}$ and 2 μM of V_{1a} **(D)**, 100 pM spectrin-actin seeds and 1.5 μM actin (10% pyrenyl-labeled). The control kinetics with 1.5 μM actin and 2 μM V_t or 1 μM $T_{\Delta 2\Delta AI}$ or 1 μM $T_{\Delta 3\Delta AI}$ or 2 μM V_{FL} or 2 μM V_{1a} are the same in all panels. **(E)** Quantification of the experiments in (A-D). **(A-E)** Fluorescence and elongation rates are expressed in arbitrary units (a.u.). Source data are provided as a Source Data file.

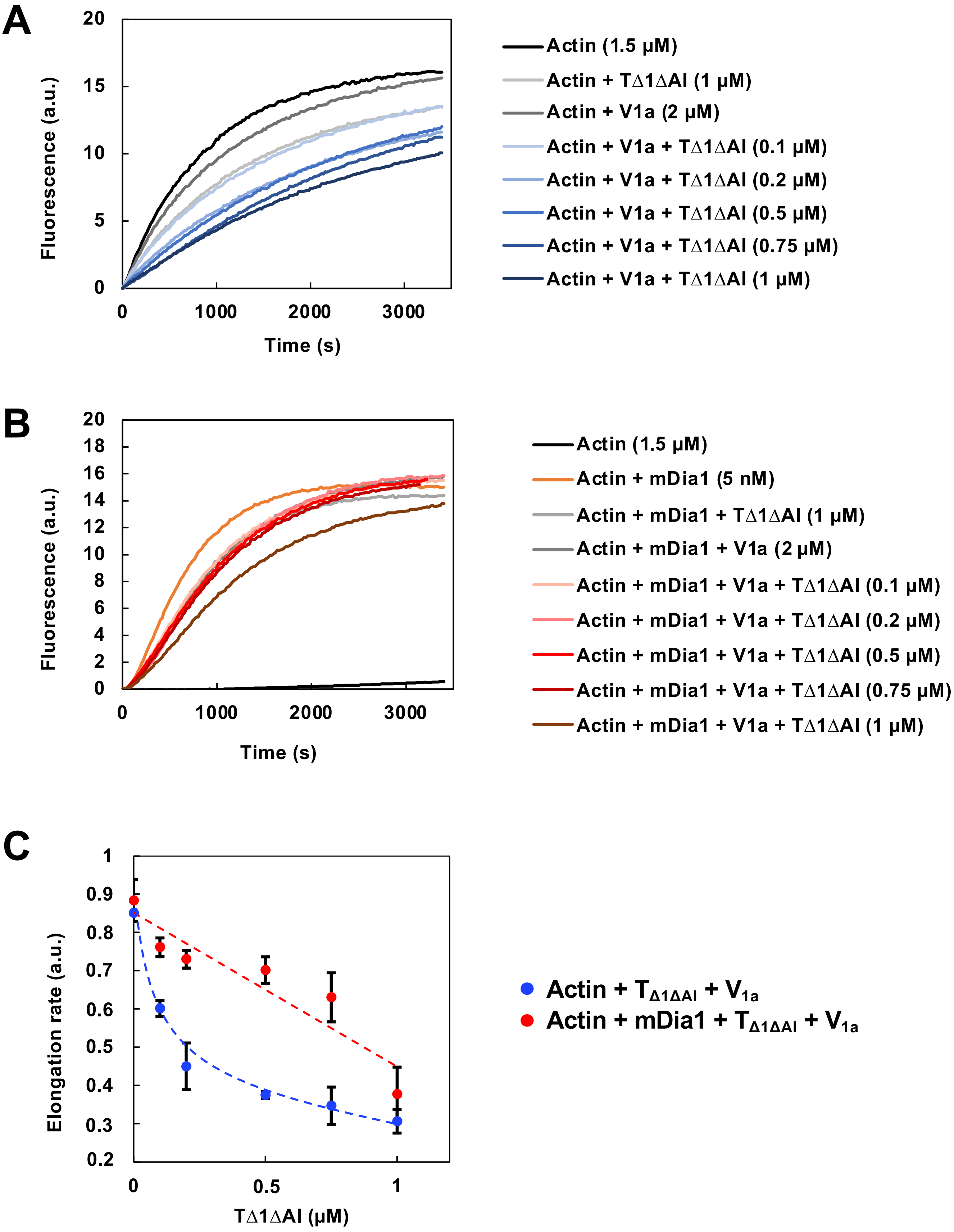

Supplementary figure 14. mDia1 antagonises the inhibition of actin filament barbed end elongation mediated by the combination of V_{1a} and $T_{\Delta1\Delta AI}$. (A, B) The elongation of actin filament barbed end was measured in the presence of 2 µM V_{1a} or 1 µM $T_{\Delta1\Delta AI}$ or 2 µM V_{1a} and increasing concentrations of $T_{\Delta1\Delta AI}$ without **(A)** and with 5 nM mDia1 **(B)**. **(C)** Kinetics from (A) and (B) are quantified to plot the maximal rate of actin elongation as a function of $T_{\Delta1\Delta AI}$ concentration. Data are mean ± SE, 3 independent experiments. **(A-C)** Fluorescence and elongation rates are expressed in arbitrary units (a.u.). Source data are provided as a Source Data file.

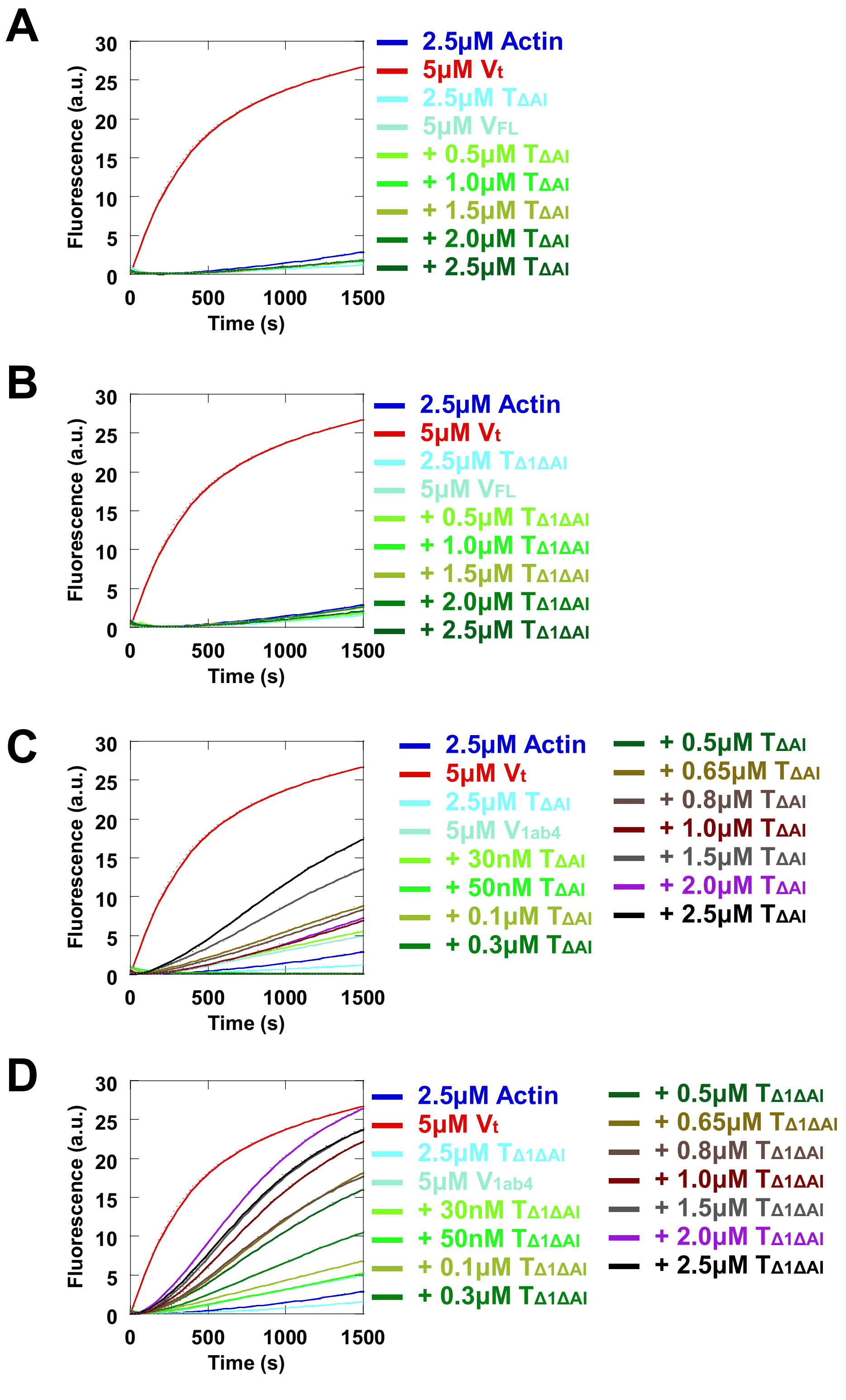

Supplementary figure 15. Talin mutants, with released autoinhibitory contacts and one exposed VBS, combine efficiently with V_{1ab4} to stimulate actin nucleation. (A-D) Actin polymerization was measured in the absence and presence of 5 µM of V_{FL} or V_{1ab4} with increasing concentrations of $T_{\Delta AI}$ or $T_{\Delta 1\Delta AI}$ and 2.5 µM G-actin (10% pyreyl-labeled) in a low salt buffer (25 mM KCl). The kinetics with 2.5 µM actin alone, used as a negative control, and 2.5 µM actin and 5 µM V_t, used as a positive control for nucleation, are the same in all panels. Fluorescence is expressed in arbitrary units (a.u.). Source data are provided as a Source Data file. **(A-D)** These experiments were repeated twice independently with the same results.

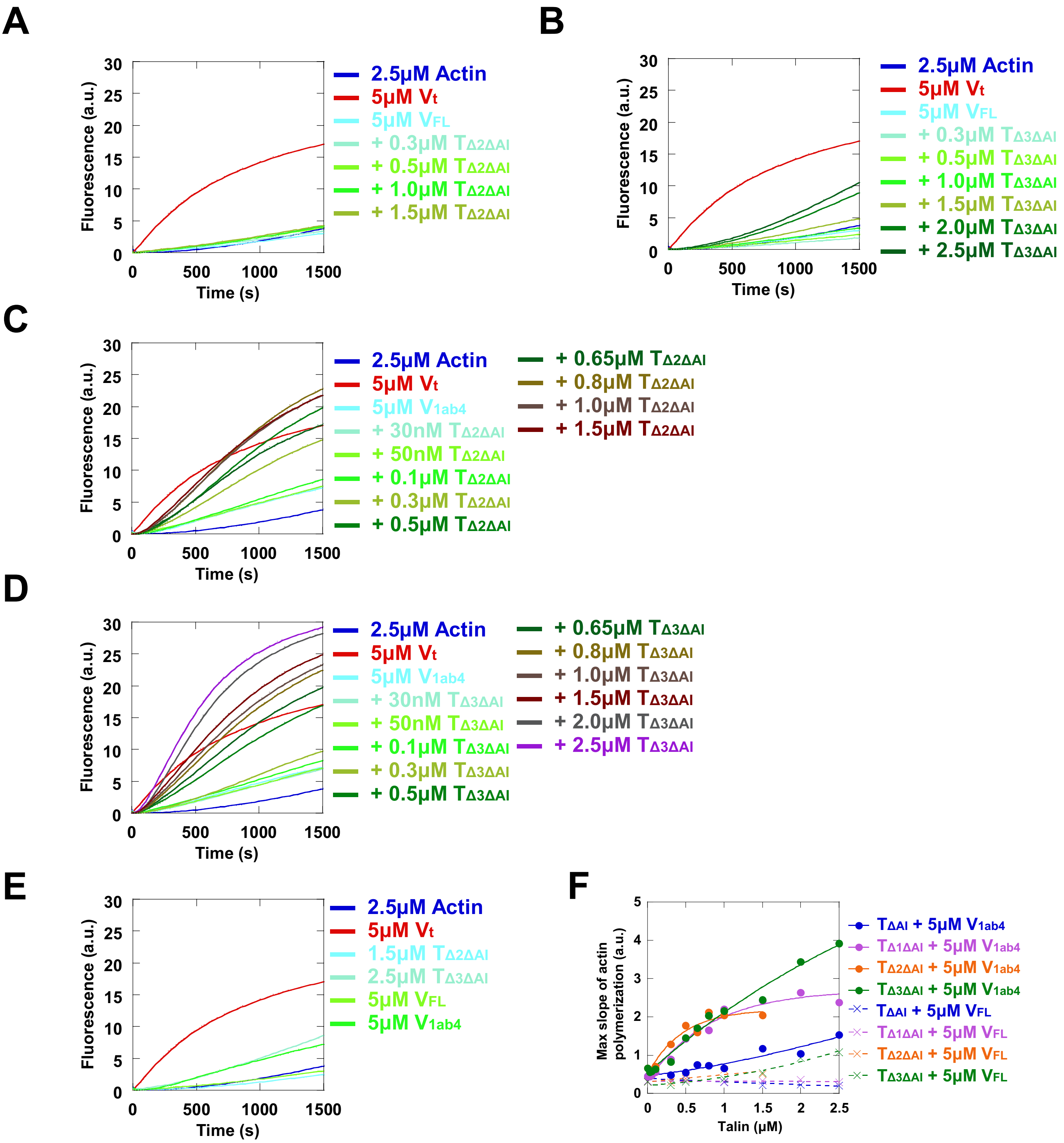

Supplementary figure 16. Talin mutants, with released autoinhibitory contacts and two or three exposed VBSs, combine efficiently with V_{1ab4} to stimulate actin nucleation. (A-D) Actin polymerization was measured in the presence of 5 µM of V_{FL} or V_{1ab4} with increasing concentrations of $T_{\Delta2\Delta AI}$ or $T_{\Delta3\Delta AI}$ and 2.5 µM actin (10% pyreyl-labeled) in a low salt buffer (25 mM KCl). **(D)** These experiments were repeated twice independently with the same results. **(E)** Additional negative control showing the polymerization of 2.5 µM actin (10% pyreyl-labeled) in the presence of 5 µM V_{FL} or 5 µM V_{1ab4} or 1.5 µM $T_{\Delta2\Delta AI}$ or 2.5 µM $T_{\Delta3\Delta AI}$. The kinetics with 2.5 µM actin alone, used as a negative control, and 2.5 µM actin and 5 µM V_t, used as a positive control for nucleation, are the same in all panels. **(F)** The maximal rate of spontaneous actin polymerization is plotted against increasing concentrations of talin mutants in the presence of V_{FL} or V_{1ab4} as described in (A-D). Data for $T_{\Delta1\Delta AI}$ + V_{FL} or V_{1ab4} and $T_{\Delta AI}$ + V_{FL} or V_{1ab4} are the same as in Figure 2D. **(A-F)** Fluorescence and actin polymerization are expressed in arbitrary units (a.u.). Source data are provided as a Source Data file..

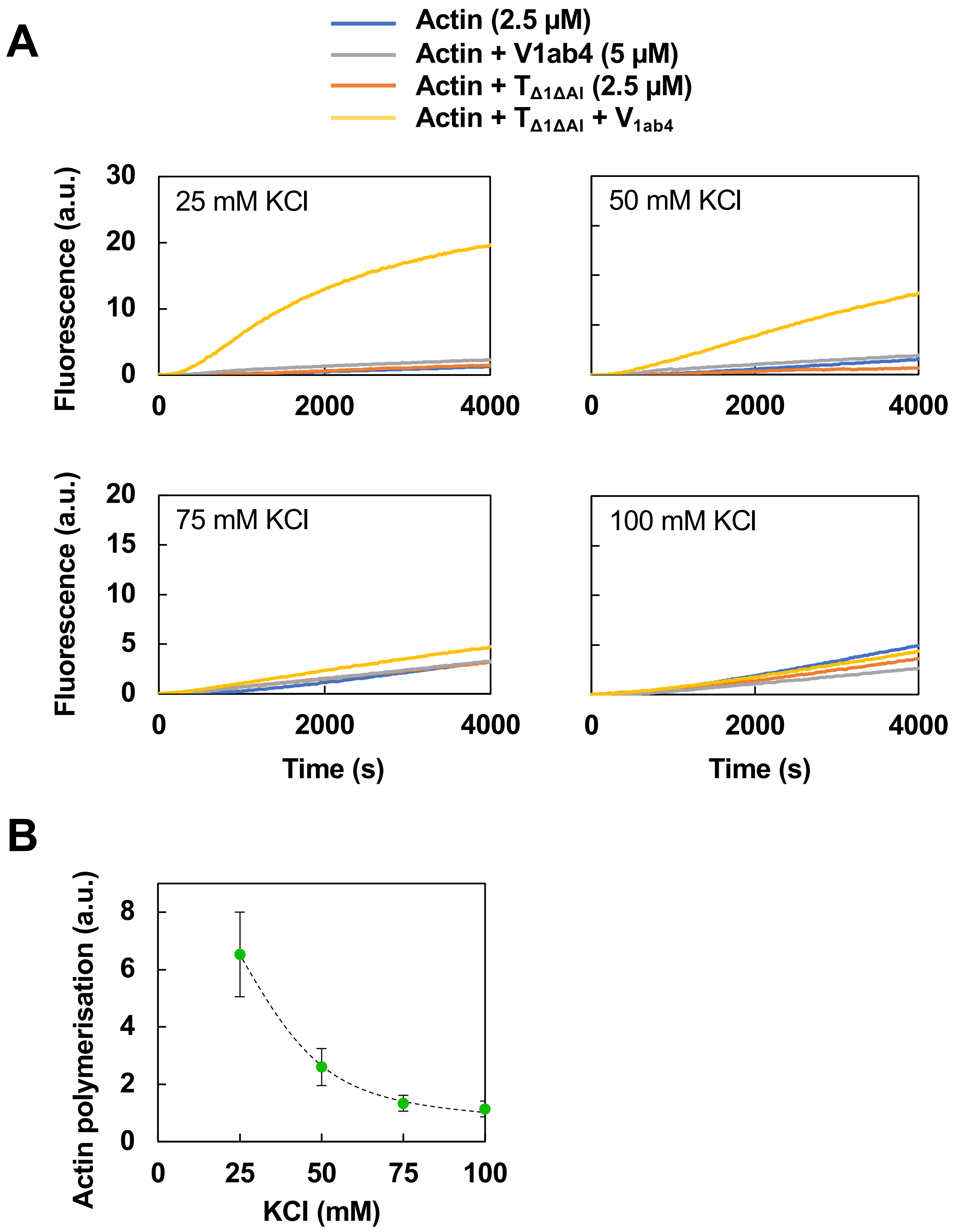

Supplementary figure 17. The stimulation of actin polymerization mediated by the combination of V_{1ab4} and $T_{\Delta1\Delta AI}$ depends on KCl concentration. (A) Actin polymerization (2.5µM G-actin, 10% pyrene-labeled) was measured alone and in the presence of 5 µM V_{1ab4}, 2.5 µM $T_{\Delta1\Delta AI}$ and both 5 µM V_{1ab4} and 2.5 µM $T_{\Delta1\Delta AI}$ at the indicated concentration of KCl. **(B)** Kinetics from (A) are quantified to plot the maximal rate of spontaneous actin polymerization as a function of KCl concentration. Data are mean ± SE, 3 independent experiments. **(A-B)** Fluorescence and actin polymerization are expressed in arbitrary units (a.u.). Source data are provided as a Source Data file.

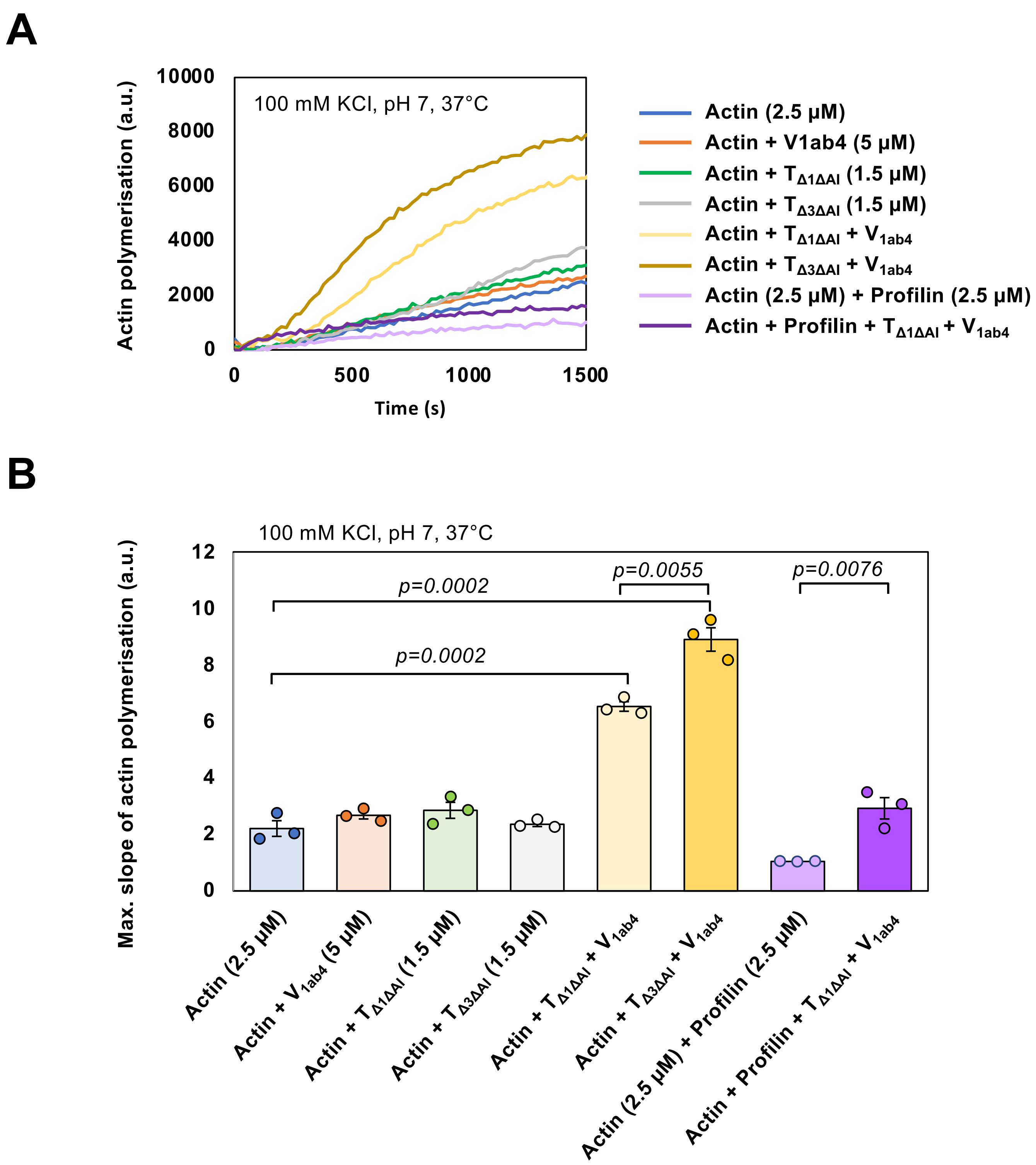

Supplementary figure 18. The stimulation of actin polymerization mediated by the combination of V_{1ab4} and $T_{\Delta1\Delta AI}$, or V_{1ab4} and $T_{\Delta3\Delta AI}$, occurs at 100 mM KCl, pH 7, 37°C. **(A)** Actin polymerization (2.5 µM G-actin, 10% pyrene-labeled) was measured alone and in the presence of 5 µM V_{1ab4} alone, 1.5 µM $T_{\Delta1\Delta AI}$ alone or 1.5 µM $T_{\Delta3\Delta AI}$ alone, and both 5 µM V_{1ab4} and 1.5 µM $T_{\Delta1\Delta AI}$ or 5 µM V_{1ab4} and 1.5 µM $T_{\Delta3\Delta AI}$ at 100 mM KCl, pH 7, 37°C. Actin polymerization (2.5 µM G-actin, 10% pyrene-labeled) was also measured in the presence of 2.5 profilin alone and in the presence of 5 µM V_{1ab4}, 1.5 µM $T_{\Delta1\Delta AI}$ and both 5 µM V_{1ab4} and 1.5 µM $T_{\Delta1\Delta AI}$. **(B)** Kinetics from (A) are quantified to plot the maximal rate of spontaneous actin polymerization at the indicated conditions. Data are mean ± SE, 3 independent experiments. Statistical analysis was performed using a two-sided unpaired t-test. **(A-B)** Fluorescence and actin polymerization are expressed in arbitrary units (a.u.). Source data are provided as a Source Data file.

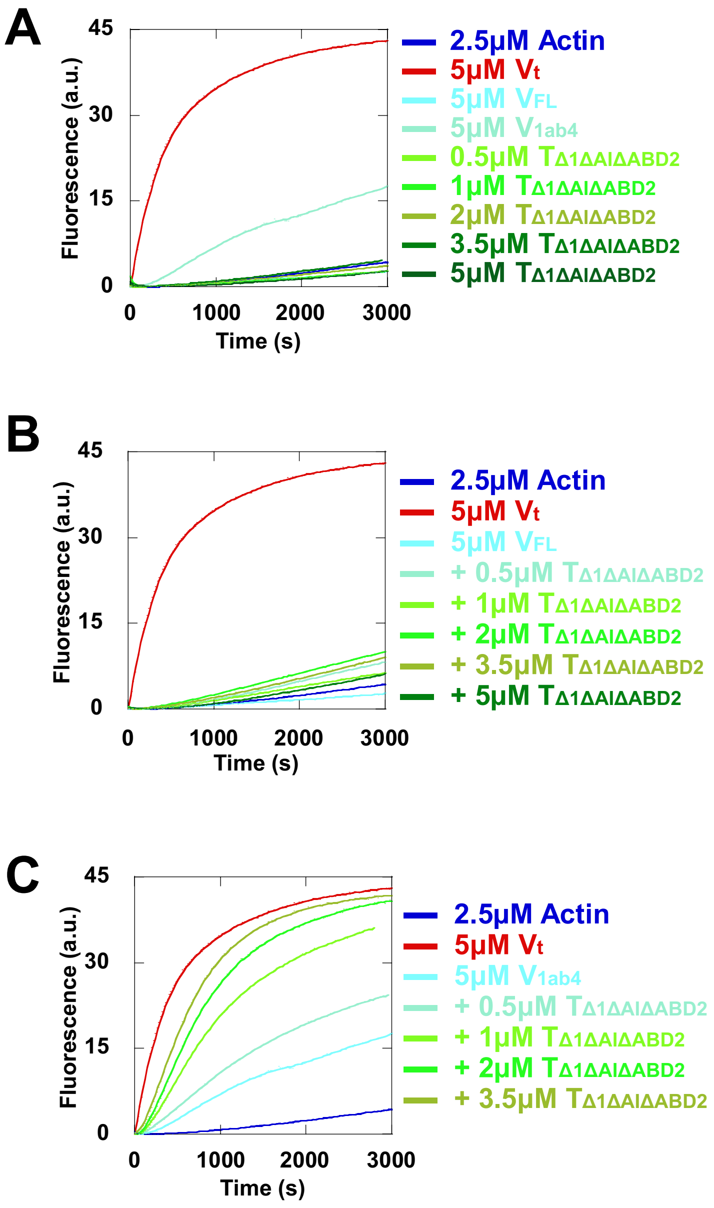

Supplementary figure 19. A talin mutant, with released autoinhibitory contacts, one exposed VBS and lacking ABD2, combines efficiently with V_{1ab4} to stimulate actin polymerization. (A-C) Spontaneous actin polymerization was measured in the presence of 2.5 µM actin (10% pyrenyl-labeled) and increasing concentrations of $T_{\Delta1\Delta AI\Delta ABD2}$ alone **(A)**, increasing concentrations of $T_{\Delta1\Delta AI\Delta ABD2}$ and 5 µM V_{FL} **(B)** and increasing concentrations of $T_{\Delta1\Delta AI\Delta ABD2}$ and 5 µM V_{1ab4} **(C)** in a low salt buffer (25 mM KCl). The kinetics with 2.5 µM actin and 5 µM V_t alone, used as a positive control or 5 µM V_{1ab4} alone are the same in all panels. Fluorescence is expressed in arbitrary units (a.u.). Source data are provided as a Source Data file. **(A-C)** These experiments were repeated twice independently with the same results.

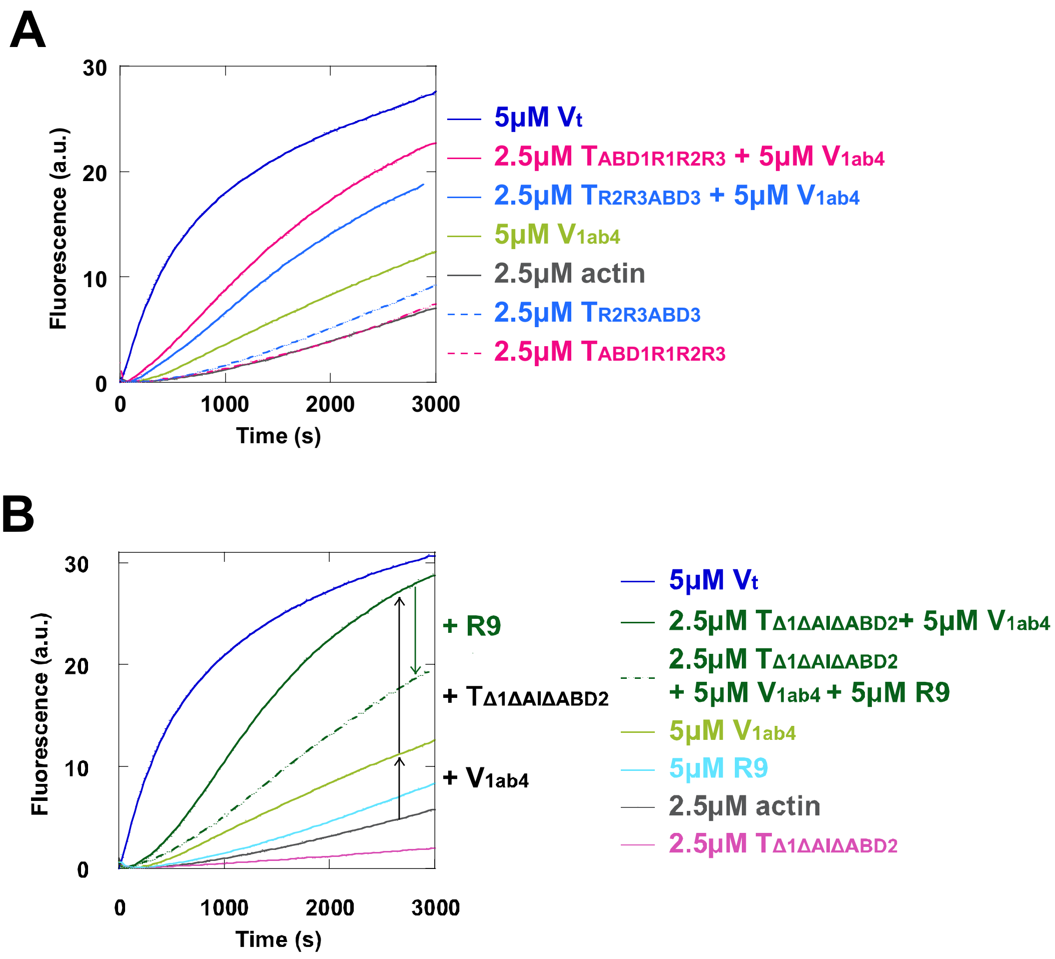

Supplementary figure 20. Importance of talin ABDs for the activities of the talin-vinculin complex. (A,B) Spontaneous actin polymerization was measured in presence of 2.5 µM actin (10% pyrenyl-labeled) and the indicated talin and vinculin mutants in a low salt buffer (25 mM KCl). The black arrows in (B) indicate the stimulation of actin polymerization by V_{1ab4} alone and V_{1ab4} + $T_{\Delta1\Delta AI\Delta ABD2}$. The dark green arrow in (B) indicates that R9 reverses the actin polymerization induced by V_{1ab4} + $T_{\Delta1\Delta AI\Delta ABD2}$. Fluorescence is expressed in arbitrary units (a.u.). Source data are provided as a Source Data file.

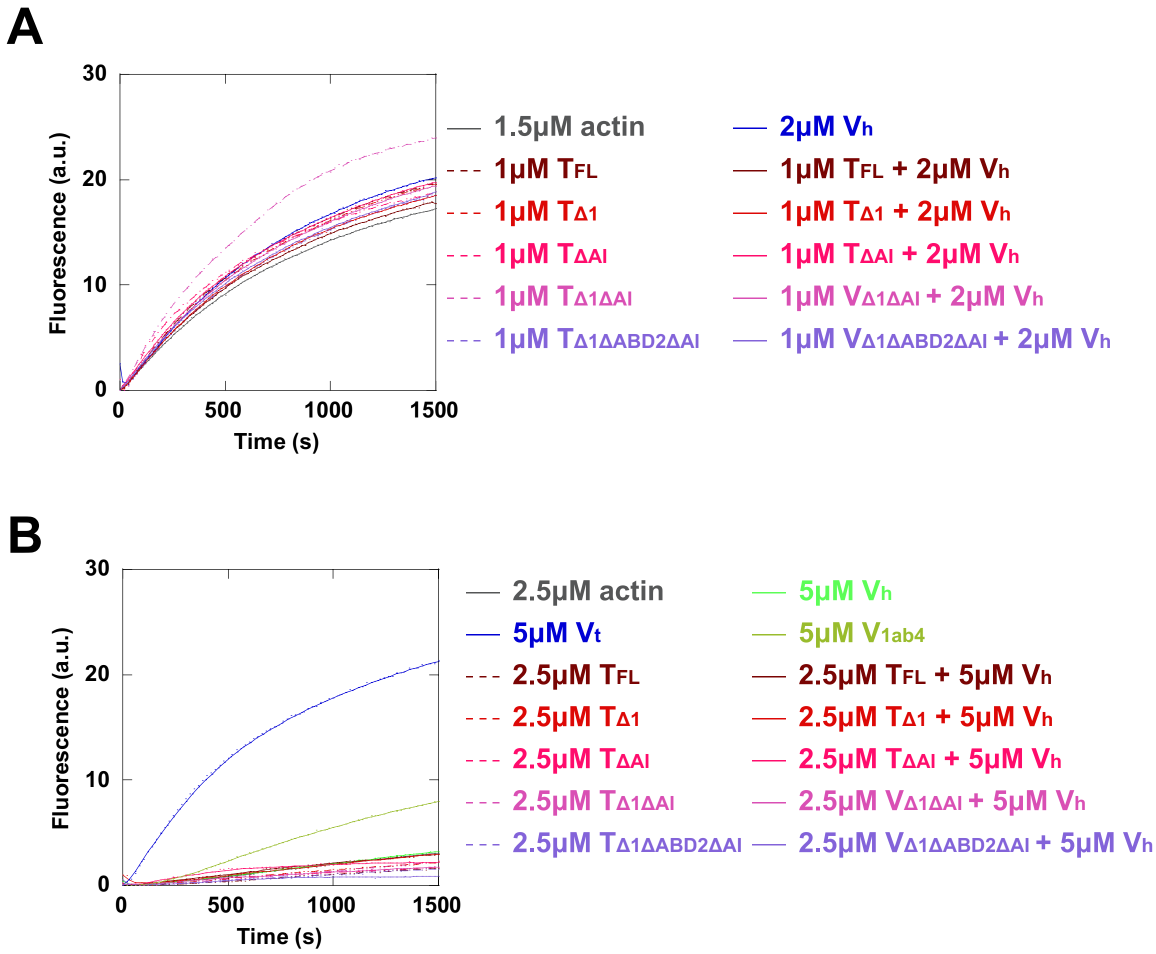

Supplementary figure 21. A talin-vinculin complex, in which vinculin lacks V_t, does not nucleate and cap actin filaments. (A) The elongation of actin filament barbed end was measured in the presence of the indicated proteins, 100 pM spectrin-actin seeds, 1.5 µM actin (10% pyrenyl-labeled). **(B)** Spontaneous actin polymerization was measured in presence of 2.5 µM actin (10% pyrenyl-labeled) and the indicated proteins in a low salt buffer (25 mM KCl). Fluorescence is expressed in arbitrary units (a.u.). Source data are provided as a Source Data file.

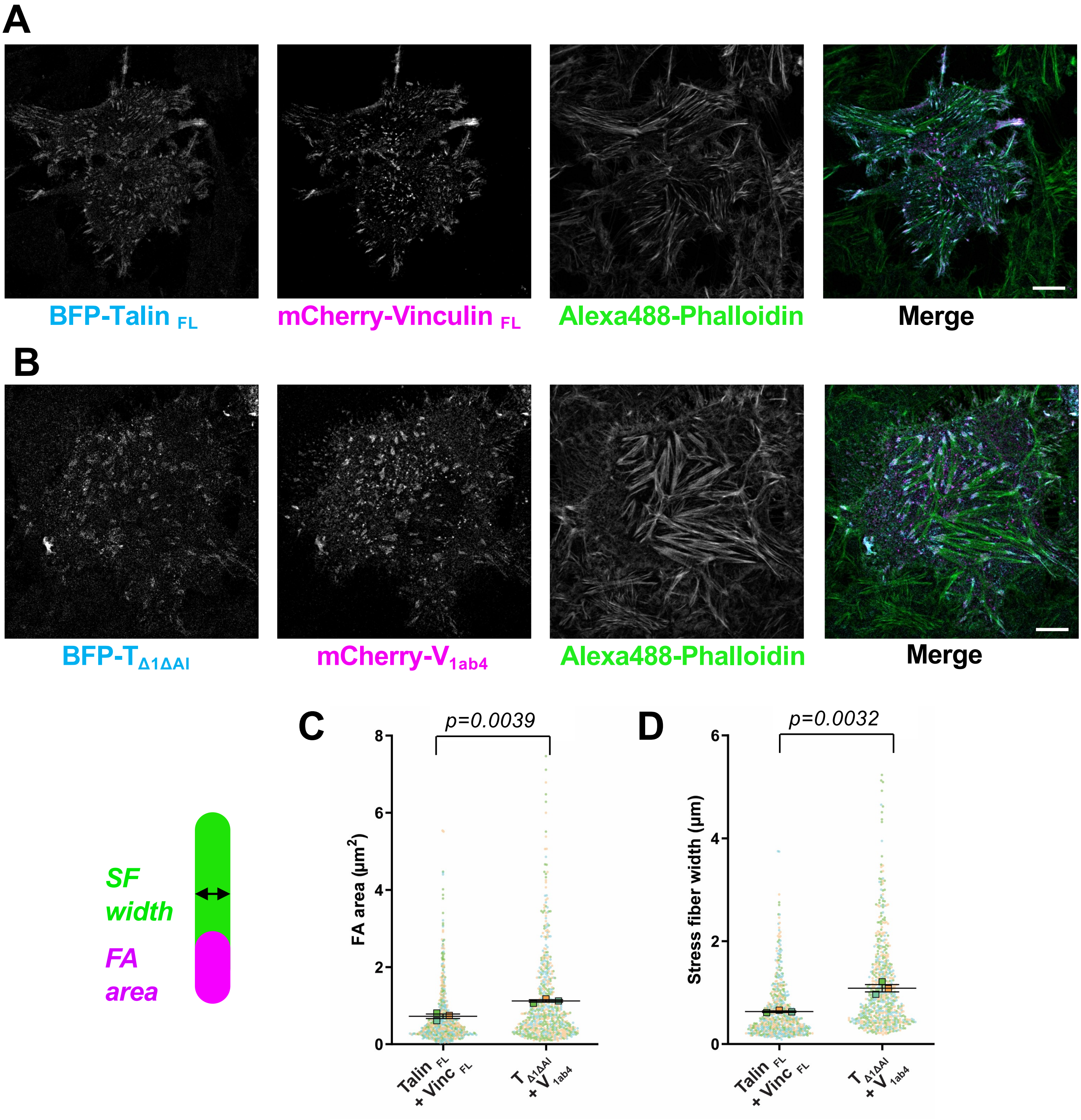

Supplementary figure 22. Expression of V_{1ab4} and $T_{\Delta1\Delta AI}$ in Hela cells induces the formation of larger focal adhesions and wider stress fibers. (A) Representative pictures of cells expressing mCherry-vinculin $_{FL}$ (magenta), BFP-talin $_{FL}$ (cyan) and stained with Alexa488-phalloidin (green) to reveal stress fibers. **(B)** Representative pictures of cells expressing mCherry-V_{1ab4} (magenta), BFP-$T_{\Delta1\Delta AI}$ (cyan) and stained with Alexa488-phalloidin (green) to reveal stress fibers. **(A, B)** scale bar is 10 µm. **(C)** Distribution of the area of focal adhesions and **(D)** distribution of the width of stress fibers in the condition mentionned in (A) and (B). **(C, D)** Experiments was performed three times. FA area and connected stress fiber width were measured from 17 (mCherry-vinculin $_{FL}$ + BFP-talin $_{FL}$) (n=6, 5, 6) cells and 16 (mCherry-V_{1ab4} + BFP-$T_{\Delta1\Delta AI}$) (n=4, 6, 6) cells. Data are mean ± sem. Each measurement represents the area of an individual FA and the width of the bound stress fiber. n= 596 (203, 224, 169) for mCherry-vinculin $_{FL}$ + BFP-talin $_{FL}$ and n=638 (259, 179, 200) for mCherry-V_{1ab4} + BFP-$T_{\Delta1\Delta AI}$. The three different colors identifiy measurements from the three different experiments. The squares framed in black are the means of these three experiments Statistical analysis was performed using a two-sided unpaired t-test. Source data are provided as a Source Data file.

Supplementary Table 1: Summary of the activities of talin and vinculin mutants and their complexes

Talin constructs							Vinculin constructs			Talin-vinculin complex activities		
Name	ABD exposed			AI released	exposed VBS in:							
	ABD1	ABD2	ABD3		R1 (1)	R3 (2)	VFL	V1a	V1ab4	Side binding	Capping	Nucleation
							x			-	+/-	-
								x		+	+/-	-
									x	+++	++	-
VBS$_1$	na	na	na	na	x		x			+		
	na	na	na	na	x			x		++		
	na	na	na	na	x				x			-
TFL (WT)			x				x				-	
								x			+	
									x			-
TΔ1			x		x		x				-	
								x			++	
									x			-
TΔ2			x			x	x				-	
								x			++	
									x			-
TΔ3			x		x	x	x				-	
								x			++	
									x			-
TΔAI	x		x	x			x				-	-
								x			++	
									x			+
TΔ1ΔAI	x		x	x	x		x				-	-
								x			+++	
									x			++
TΔ2ΔAI	x		x	x		x	x				++	-
								x			+++	
									x			++
TΔ3ΔAI	x		x	x	x	x	x				++	+
								x			+++	
									x			++
TΔ1ΔAIΔABD2	x		x	x	x		x				-	-
								x			+++	
									x			++

Supplementary Table 2. Primer sequences for the constructs used in the study

Primer name	Primer description	Sequence (5' → 3')
Ta-196-Bam-CLC	Forward primer for pGEX6P1-$T_{F2F3R1R2R3}$	CGGGCGGATCCAAGTTCTTTTACTCAGACCAGAAT
3-primer-911-EcorI	Reverse primer for pGEX6P1-$T_{F2F3R1R2R3}$	TTTTTGAATTCTCAGTGGTGGTAGTAGTGGTAACCTCCT TTCTTGATGGCATTCTGCGCAGCTG
o217	Forward primer for R2R3 of pETM $T_{R2R3ABD3}$	GGCGGTACCATTGGGGAAAGTGATACTGAC
o218	Reverse primer for R2R3 of pETM $T_{R2R3ABD3}$	GGCGGATCCACCGGATTTCTTGATGGCATTCTG
o220	Forward primer for R13 of pETM $T_{R2R3ABD3}$	GGCGGATCCGGTGGAGACCCCACAGTCATTGCTG
o221	Reverse primer for R13 of pETM $T_{R2R3ABD3}$	GGCGAATTCTTAATGGTGATGGTGATGATGAC
o174	Forward primer for the first step of pETM $T_{\Delta 1\Delta AI\Delta ABD2}$	GGCGGCGCTAGCCCGTTCTCTCAAGACTTACGGTGTC
o224	Reverse primer for the first step of pETM $T_{\Delta 1\Delta AI\Delta ABD2}$	GGCCCCGGGCTCAGCACTGGCTGGTTG
o225	Forward primer for the second step of pETM $T_{\Delta 1\Delta AI\Delta ABD2}$	GGCCCCGGGGAAAGTGATACTGACCCCCACTTC
o228	Reverse primer for the second step of pETM $T_{\Delta 1\Delta AI\Delta ABD2}$	GGCCCATGGAGCTAAAGATGTTC
Backbone-TagBFP-fwd	Forward primer for BFP of pBFP-Talin $_{FL}$ and pBFP-$T_{\Delta 1\Delta AI}$	CTACGTGAGGTACCGCGGGCCCGGGATC
Backbone-TagBFP-rev	Reverse primer for BFP of pBFP-Talin $_{FL}$ and pBFP-$T_{\Delta 1\Delta AI}$	GTCGACTGCAGAATTCGAAGCTTGAGC
Insert-Talin-fwd	Forward primer for talin of pBFP-Talin $_{FL}$ and pBFP-$T_{\Delta 1\Delta AI}$	GAATTCTGCAGTCGACATGGTTGCACTTTCACTGAAGA TCAGC
Insert-Talin-rev	Reverse primer for talin of pBFP-Talin $_{FL}$ and pBFP-$T_{\Delta 1\Delta AI}$	GCCCGCGGTACCTCACGTAGAATCGAGACCGAGGAG
Backbone-mCherry-fwd	Forward primer for mCherry of pmCherry-Vinculin$_{FL}$ and pmCherry-V_{1ab4}	GTCGACGGATCCACCGGATCTAGATAACTGATCAT
Backbone-mCherry-rev	Reverse primer for mCherry of pmCherry-Vinculin$_{FL}$ and pmCherry-V_{1ab4}	CACTGGTGCCATACTAGTGAGTCCGGACTTGTACAGCT CG
Insert-Vinculin-fwd	Forward primer for vinculin of pmCherry-Vinculin$_{FL}$ and pmCherry-V_{1ab4}	ACTAGTATGGCACCAGTGTTTCATACGCG
Insert-Vinculin-rev	Reverse primer for vinculin of pmCherry-Vinculin$_{FL}$ and pmCherry-V_{1ab4}	CGGTGGATCCGTCGACCTACTGGTACCAGGGAGTCTTT CTAACCC
