# Supplementary material for: Talin and vinculin combine their activities to trigger actin assembly
Source: Nat Commun. 2024 Nov 3;15:9497. doi: 10.1038/s41467-024-53859-1 (PMC11532549; doi:10.1038/s41467-024-53859-1)
Supplement: Supplementary file 3 — Description of Additional Supplementary Files [file 41467_2024_53859_MOESM3_ESM.pdf]

Supplementary Movie 1. Observation of the activity of $T_{\Delta1\Delta AI}$ and V_{1ab4} on single actin filaments in TIRF microscopy. Conditions: 1 µM actin (5% Alexa 488-labeled) in 5 mM Tris, pH 7.8, 200 µM ATP, 1% methylcellulose, 5 mM 1,4-diazabicyclo(2,2,2)-octane (DABCO), 100 mM KCl, 1 mM $MgCl_2$, 200 µM EGTA, 40 mM DTT supplemented with 2.5 µM $T_{\Delta1\Delta AI}$ or 5 µM V_{1ab4} or 2.5 µM $T_{\Delta1\Delta AI}$ and 5 µM V_{1ab4}. Scale bar = 15 µm.

Supplementary Movie 2. Observation of the activity of $T_{\Delta1\Delta AI}$ and V_{1ab4} on single actin filaments in TIRF microscopy. Conditions: 1 µM actin (5% Alexa 488-labeled) in 5 mM Tris, pH 7.8, 200 µM ATP, 1% methylcellulose, 5 mM 1,4-diazabicyclo(2,2,2)-octane (DABCO), 25 mM KCl, 1 mM $MgCl_2$, 200 µM EGTA, 40 mM DTT supplemented with 2.5 µM $T_{\Delta1\Delta AI}$ or 5 µM V_{1ab4} or 2.5 µM $T_{\Delta1\Delta AI}$ and 5 µM V_{1ab4}. Scale bar = 15 µm.

Supplementary Movie 3. Observation of the activity of $T_{\Delta1\Delta AI\Delta ABD2}$ and V_{1ab4} on single actin filaments in TIRF microscopy. Conditions: 1 µM actin (5% Alexa 488-labelled) in 5 mM Tris, pH 7.8, 200 µM ATP, 1% methylcellulose, 5 mM 1,4-diazabicyclo(2,2,2)-octane (DABCO), 25 mM KCl, 1 mM $MgCl_2$, 200 µM EGTA, 40 mM DTT supplemented with 2.5 µM $T_{\Delta1\Delta AI\Delta ABD2}$ or 5 µM V_{1ab4} or 2.5 µM $T_{\Delta1\Delta AI\Delta ABD2}$ and 5 µM V_{1ab4}. Scale bar = 15 µm.

Supplementary Movie 4. Observation of talin, vinculin and actin during the nucleation of a single actin filament. 0.2 µM $T_{\Delta1\Delta AI}$ (78% Alexa594-labelled) and 1 µM V_{1ab4} (18% Alexa647-labelled) were first mixed, diluted 20 times to reach final concentrations of 10 nM $T_{\Delta1\Delta AI}$ and 50 nM V_{1ab4}, injected on a flow chamber to immobilize $T_{\Delta1\Delta AI}$-V_{1ab4} complexes non-specifically on the surface, and finally supplemented with 0.8 µM actin (5% Alexa488-labelled).

Supplementary Movie 5. Recruitment of actin by a talin-vinculin complex followed by the elongation of a filament after a delay. 0.2 µM $T_{\Delta1\Delta AI}$ (78% Alexa594-labelled) and 1 µM V_{1ab4} (18% Alexa647-labelled) were first mixed, diluted 20 times to reach final concentrations of 10 nM $T_{\Delta1\Delta AI}$ and 50 nM V_{1ab4}, injected on a flow chamber to immobilize $T_{\Delta1\Delta AI}$-V_{1ab4} complexes non-specifically on the surface, and finally supplemented with 0.8 µM actin (5% Alexa488-labelled).

Supplementary Movie 6. Capture and barbed-end capping of an existing filament by a talin-vinculin complex. 0.2 µM $T_{\Delta1\Delta AI}$ (78% Alexa594-labelled) and 1 µM V_{1ab4} (18% Alexa647-labelled) were first mixed, diluted 20 times to reach final concentrations of 10 nM $T_{\Delta1\Delta AI}$ and 50 nM V_{1ab4}, injected on a flow chamber to immobilize $T_{\Delta1\Delta AI}$-V_{1ab4}

complexes non-specifically on the surface, and finally supplemented with 0.8 µM actin (5% Alexa488-labelled).

Supplementary Movie 7. Transient barbed-end capping of a filament by a talin-vinculin complex followed by its release. 0.2 µM $T_{\Delta1\Delta AI}$ (78% Alexa594-labelled) and 1 µM V_{1ab4} (18% Alexa647-labelled) were first mixed, diluted 20 times to reach final concentrations of 10 nM $T_{\Delta1\Delta AI}$ and 50 nM V_{1ab4}, injected on a flow chamber to immobilize $T_{\Delta1\Delta AI}$-V_{1ab4} complexes non-specifically on the surface, and finally supplemented with 0.8 µM actin (5% Alexa488-labelled).

Supplementary Movie 8. FRAP in Hela cells expressing EGFP-actin together with mCherry-Vinculin $_{FL}$ and BFP-Talin $_{FL}$. The movie shows mCherry-vinculin $_{FL}$ and EGFP-actin recovery after bleaching. Note that only mCherry-Vinculin $_{FL}$ and EGFP-actin have been imaged after bleaching to reduce time intervals.

Supplementary Movie 9. FRAP in Hela cells expressing EGFP-actin together with mCherry-V_{1ab4} and BFP-$T_{\Delta1\Delta AI}$. The movie shows mCherry-V_{1ab4} and EGFP-actin recovery after bleaching. Note that only mCherry-V_{1ab4} and EGFP-actin have been imaged after bleaching to reduce time intervals.
